# Destabilization of mutualistic interactions shapes the early heat stress response of the coral holobiont

**DOI:** 10.1186/s40168-024-02006-5

**Published:** 2025-01-31

**Authors:** Emma Marangon, Nils Rädecker, Joan Y. Q. Li, Marko Terzin, Patrick Buerger, Nicole S. Webster, David G. Bourne, Patrick W. Laffy

**Affiliations:** 1 Australian Institute of Marine Science, Townsville, QLD Australia; 2 College of Science and Engineering, James Cook University, Townsville, QLD Australia; 3 AIMS@JCU, Townsville, QLD Australia; 4 Laboratory for Biological Geochemistry, School of Architecture, Civil and Environmental Engineering, École Polytechnique Fédérale de Lausanne, Lausanne, Switzerland; 5 Applied Biosciences, Macquarie University, North Ryde, NSW Australia; 6 Australian Centre for Ecogenomics, University of Queensland, Brisbane, QLD Australia; 7 Institute for Marine and Antarctic Studies, University of Tasmania, Hobart, TAS Australia

**Keywords:** Coral, Heat stress, Symbiodiniaceae, Microbiome, Gene expression profiling, Transcriptomics, Physiology, Holobiont, Symbiosis

## Abstract

**Background:**

The stability of the symbiotic relationship between coral and their dinoflagellate algae (Symbiodiniaceae) is disrupted by ocean warming. Although the coral thermal response depends on the complex interactions between host, Symbiodiniaceae and prokaryotes, the mechanisms underlying the initial destabilization of these symbioses are poorly understood.

**Results:**

In a 2-month manipulative experiment, we exposed the coral *Porites lutea* to gradually increasing temperatures corresponding to 0–8 degree heating weeks (DHW) and assessed the response of the coral holobiont using coral and Symbiodiniaceae transcriptomics, microbial 16S rRNA gene sequencing and physiological measurements. From early stages of heat stress (< 1 DHW), the increase in metabolic turnover shifted the holobiont to a net heterotrophic state in which algal-derived nutrients were insufficient to meet host energy demands, resulting in reduced holobiont performance at 1 DHW. We postulate the altered nutrient cycling also affected the coral-associated microbial community, with the relative abundance of *Endozoicomonas* bacteria declining under increasing heat stress. Integration of holobiont stress responses correlated this decline to an increase in expression of a host ADP-ribosylation factor, suggesting that Symbiodiniaceae and *Endozoicomonas* may underlie similar endosymbiotic regulatory processes.

**Conclusions:**

The thermotolerance of coral holobionts therefore is influenced by the nutritional status of its members and their interactions, and this identified metabolic interdependency highlights the importance of applying an integrative approach to guide coral reef conservation efforts.

Video Abstract

**Supplementary Information:**

The online version contains supplementary material available at 10.1186/s40168-024-02006-5.

## Background

Climate change and other anthropogenic stressors are driving a decline of coral reefs worldwide [1]. Increasing sea surface temperatures trigger coral bleaching (loss of endosymbiotic dinoflagellates of the family Symbiodiniaceae), often resulting in widespread coral mortality [2]. Given the increased frequency and severity of bleaching events in the last few decades [3], a greater focus on characterizing the mechanisms underlying bleaching susceptibility is required to support effective reef conservation strategies. Coral responses to heat stress are complex and influenced by multiple factors, including the interplay between the coral host and its associated symbionts [4–6]. The exchange and recycling of limiting nutrients between Symbiodiniaceae and their host are critical for the ecological success of corals in oligotrophic environments [7]. Altered nutrient cycling (especially carbon and nitrogen) may thus be a major driver of the initial destabilization of the coral symbiosis under high temperatures [6, 8, 9]. In addition, bacteria and archaea (i.e. ‘microbes’ hereafter) also contribute to holobiont functioning, for example in the form of nutrient transformation and antimicrobial activity, and may therefore influence coral thermotolerance [4, 10–12]. However, our understanding of the ecological and metabolic processes initiating the destabilization of these symbiotic relationships under heat stress is still incomplete. Elucidating and integrating the response of each specific holobiont member (i.e. host, Symbiodiniaceae, microbes) to increasing temperatures is therefore critical for deciphering the mechanisms underlying coral holobiont breakdown.

Stress intensity strongly influences the coral holobiont response [13, 14]. Transcriptomic analyses of heat-stressed corals revealed that immunity responses, cytoskeleton organization, antioxidant expression, heat-shock proteins and pre-apoptotic responses are among the most commonly enriched pathways under high temperatures and have been linked to severe stress responses [5]. However, mechanisms underlying the initial destabilization of the symbiosis are highly debated. The leading hypothesis stating that the production of reactive oxygen species (ROS) by the Symbiodiniaceae triggers host immune response, and in turn, symbiosis breakdown [7], has been recently challenged [15–18]. Growing evidence suggests that nitrogen availability may play a central role in the regulation of coral symbiosis [6, 9, 19, 20]. In a healthy state, nitrogen limitation within the holobiont induces Symbiodiniaceae to release the excess photosynthates (e.g. glucose) to the host, fuelling host respiration and efficient carbon recycling [21–23]. Heat stress, however, disrupts this fine equilibrium by enhancing energy demand and catabolic ammonium production in the host metabolism [6]. Increased nitrogen availability within the holobiont may thus stimulate Symbiodiniaceae to proliferate and retain photosynthates, thereby destabilizing the ecological basis of the mutualism [6, 23].

Adding to this complexity, microbial communities may affect the thermotolerance of their cnidarian hosts [10, 24, 25]. Indeed, bacterial metabolic capabilities may represent a key advantage under heat stress providing potential alternative metabolic pathways to maintain coral holobiont functioning [4]. Coral-associated microbes may also affect the stability of the coral-Symbiodiniaceae symbiosis, altering antioxidant capacity (e.g. through DMSP cycling) or nutrient levels (e.g. nitrogen transformation) [26–28]. For example, elevated nitrogen availability within the coral *Stylophora pistillata* under heat stress alleviates the host and symbionts’ metabolic dependency on nitrogen-fixing bacteria, driving compositional and functional changes in the diazotrophic microbial community [6, 29]. Integrating microbial community dynamics within the holobiont is thus required to understand the fundamental mechanisms of coral stress responses.

**Fig. 1:**
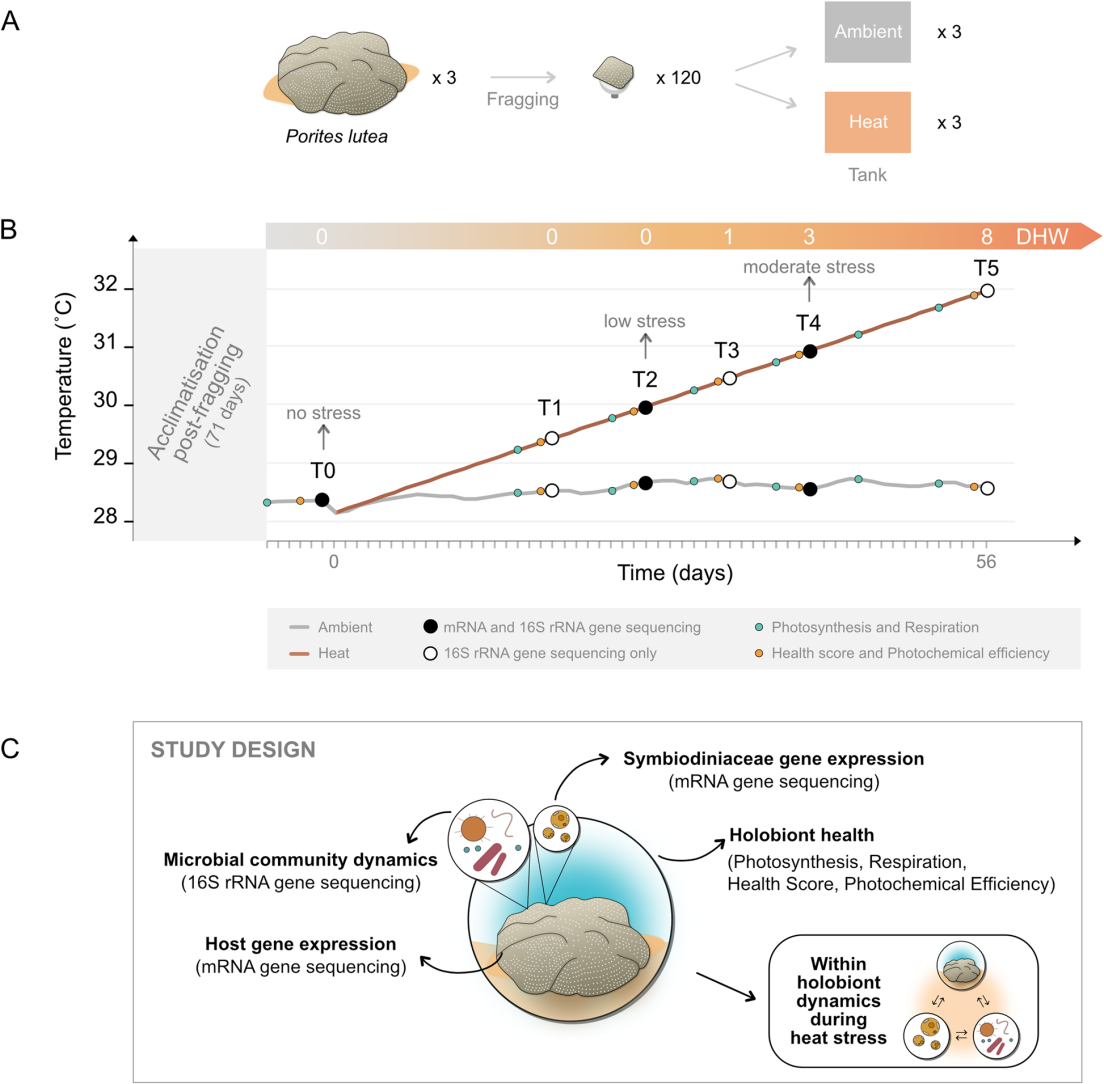
Experimental design of the 2-month incremental heat stress experiment on the thermal-tolerant coral *P. lutea*. **A** Three coral colonies were collected from Pelorus Island (central GBR), fragged into smaller pieces and randomly distributed across six tanks (ambient and heat treatment). **B** For determining Symbiodiniaceae, host and microbial responses to increasing temperatures, coral fragments were sampled at T0–T5 for mRNA and/or 16S rRNA gene sequence analyses. Degree heating weeks (DHW), representing the accumulated heat stress, are shown for each sampling time. Health score, net photosynthesis, respiration and photochemical effective efficiency were assessed over time as proxy for host health. **C** Overview of the sampling design

Here, we combined physiological and molecular tools (mRNA and 16S rRNA gene sequencing) to characterize the relationship among the thermotolerant coral *Porites lutea* host, Symbiodiniaceae and associated microbes under gradually increasing temperatures (28–32 °C; equivalent to 0–8 degree heating weeks) over a 56-day experiment (Fig. 1). This manipulative heat stress experiment (i) characterizes the temporal cascade of responses in host and Symbiodiniaceae gene expression as well as microbial community structure with increasing stress intensity, (ii) links the individual changes in the response of holobiont members to the physiological performance of the holobiont, and (iii) integrates holobiont member responses to early heat stress by identifying the main correlations between changes in host and Symbiodiniaceae gene expression and microbial community structure.

## Methods

### Coral collection and experimental design

Three colonies of the reef-building coral *P. lutea* were collected (~ 5-m depth) from Pelorus Island (central GBR, Australia) in September 2019 (permit G12/35236.1). Colonies were transferred to the National Sea Simulator (SeaSim) at the Australian Institute of Marine Science (Townsville, Australia), where they were maintained outdoors in independent flow-through tanks at ambient temperature for 3 weeks. Following the acclimation period, each colony was sampled for 16S rRNA gene sequencing (‘baseline’ samples; *n* = 4, with two samples collected from colony A), and fragmented into at least 38 pieces (approximately 4 × 4 cm, total of 120 coral fragments) using an underwater drill, ring saw and band saw. Fragments were glued onto aragonite plugs and transferred to indoor flow-through tanks (*n* = 6; 50L; 0.8 L/min), distributing 5–7 fragments from each colony of origin per tank. Coral fragments were maintained at ambient temperature for 10 weeks, with temperatures reflecting the average daily temperature of the central GBR (Davies Reef, 1991 – 2012; 27 ± 0.8 ˚C; [30]). During this period, corals were fed daily with microalgae (*Nannochloropsis oceanica*, *Isochrysis* sp., *Chaetoceros muelleri*, *Dunaliella* sp., *Proteomonas sulcata*; 2000 cells/mL in tank) and rotifers (480 rotifers/L in tank).

Following the acclimation period, coral fragments under the heat treatment were experimentally exposed to incremental temperatures for 56 days (0.07 °C daily increment, from 28.16 to 32.01 °C corresponding to 0–8 degree heating week; *n* = 3 tanks), while ambient conditions mimicked daily natural fluctuations in summer temperatures in the central GBR ([30], 28.5 ± 0.12 °C; *n* = 3 tanks). Seawater temperatures in both ambient and heat tanks were regulated by a computer-controlled system which provides fine scale control of environmental parameters and further stabilized through water jackets. A natural 12:12-h light:dark cycle was set up using SOL LED lights (AquaIllumination) with 4-h ramping at sunrise and sunset (max. light intensity 130 µmol photons m^−2^ s^−1^), and corals were fed with microalgae and rotifers three times per week.

During the experiment, four physiological parameters were regularly measured to assess holobiont health: photosynthesis and respiration rates, photochemical efficiency and health score (Fig. 1). In parallel, sampling was performed at six time points (weeks 0, 3, 4, 5, 6, 8 hereafter referred to as T0–T5) by collecting one fragment of each colony of origin per tank for mRNA and 16S rRNA gene sequencing (Fig. 1). Each fragment was cut into smaller pieces using a sterile band saw, rinsed with filtered seawater (0.22 µL), snap frozen in liquid nitrogen and stored at − 80 °C until further processing. Seawater was also sampled at T0, T3 and T5 to provide an environmental control for 16S rRNA gene analyses; 1 L of seawater per each tank was filtered through 0.22-µL Sterivex filters (Millipore), which were subsequently stored at − 80 °C until DNA extraction. Additionally, microalgae and rotifers used to feed the corals were sampled for 16S rRNA gene analyses to control for diet-introduced microbes in the coral microbiome.

### Holobiont physiological health metrics

Coral health was regularly assessed using the CoralWatch Coral Health Chart [31] to detect visible signs of bleaching (reduction in pigmentation). Pictures of the coral fragments were taken under constant settings and analysed in ImageJ [32], where the mean grey value for each fragment (based on *n* = 5 measurements) was standardized to the CoralWatch D1–D6 references using linear regressions to determine a comparable health score [33]. Health score was measured on each coral fragment over time (T0: *n* = 60 per treatment; T1: *n* = 49–52 per treatment; T2: *n* = 42 per treatment; T3: *n* = 32–33 per treatment; T4: 23–24 per treatment; T5: 13–15 per treatment).

Net photosynthesis (oxygen production, P) and respiration (oxygen consumption, R) rates were measured to estimate the carbon flux between host and Symbiodiniaceae through the photosynthesis-to-respiration ratio (P/R) and were performed using a modified process previously described in Schoepf et al. [34]. Briefly, dissolved oxygen was measured using an optical probe after placing coral fragments (T0–T4: *n* = 18 per treatment; T5: *n* = 13–15 per treatment) in 600-mL plastic chambers for 50-min incubation under constant light intensity (130 µmol photons m^−2^ s^−1^) and 75-min incubation in dark conditions, respectively. Corals were acclimated for at least 1 h to the respective light or dark conditions prior to incubation. During the incubation period, chambers were placed in water baths with temperature reflecting treatment conditions, and water motion within each chamber was generated by a magnetic stirrer. To account for the background variation in oxygen flux, chambers containing only seawater were incubated alongside the coral fragments (i.e. blanks; *n* = 2 per tank/time point). P and R rates were adjusted by the oxygen concentration in the blanks and standardized by the volume of water in each chamber and the surface area of the fragment. The water volume was calculated using the displacement method, while each fragment’s surface area was estimated via the aluminium foil method [35]. P/R was calculated as ratio of 12 h of gross P (= net P + R) and 24 h of R [34]. Notably, this approach likely overestimates daily gross photosynthesis as the ramping of light intensities at sunrise, and sunset is not accounted for in the analyses. While these P/R ratios may not provide an accurate assessment of daily carbon budgets, they do provide a robust estimate of relative changes between treatments and facilitate comparison with other studies.

To assess the photosynthetic efficiency of coral-associated Symbiodiniaceae under increasing temperatures, photochemical effective efficiency (∆F/Fm′) was analysed [36]. Briefly, ∆F/Fm′ was measured using a Mini-PAM fluorometer (Walz, Germany; settings: *MI* = 4; *SI* = 8; *SW* = 0.8; *G* = 2; *D* = 2) on corals acclimated to peak light conditions for at least 1 h. Measurements (*n* = 3 per fragment at each time point; all fragments were assessed) were taken at a constant distance of 0.9 cm from the coral tissue, and the mean photochemical effective efficiency was calculated per fragment at each time point.

The effect of incremental temperatures on physiological host health metrics was tested using generalized linear mixed models (‘glmmTMB’ package; [37]) in R (version 4.0.3; [38]). Specifically, the effect of treatment and time on health score and photosynthesis rates (square-root transformed) were analysed with a Gaussian model, respiration rates (absolute value) as well as P/R ratio with a gamma distribution and photochemical effective efficiency (square-root transformed) with a beta distribution. Data transformation was performed to meet model assumptions. For testing net P rates, an offset was applied to the data to remove negative values prior to the square-root transformation. In the models, colony of origin was also included as a fixed effect, while tank and fragment were included to account for repeated measures. Model assumptions were checked with ‘DHARMa’ [39], and model selection was completed based on the corrected Akaike information criterion (AICc) when more than one model distribution was appropriate for the analyses. Pairwise comparisons were run using ‘emmeans’ [40] with Šidàk correction.

### Coral host and Symbiodiniaceae transcriptomics

After crushing the fragments at low temperatures using a French press, RNA was isolated using the PureLink™ RNA Mini Kit (Thermo Fisher Scientific), including a TRIzol/chloroform step. First, coral samples (tissue and skeleton) were added to bead tubes (MP; *n* = 2 tubes per sample) containing 1-mL TRIzol and then homogenized using the FastPrep-24 5G instrument (MP Biomedicals). After spinning down the homogenized tissue, the supernatant was mixed with 200-µL chloroform and centrifuged, and the upper phase containing RNA was then purified following the manufacturer’s protocol, which included an on-column DNase treatment (Thermo Fisher Scientific). Purified RNA was diluted in 34 µL of nuclease-free water, and RNA quality and concentration were assessed using a NanoDrop 2000 spectrophotometer (Thermo Scientific) and Agilent 2100 Bioanalyzer with the RNA 6000 Pico assay. High-quality RNA was stored at − 80 °C until submission to the Ramaciotti Centre for Genomics (University of New South Wales, Australia) for Illumina-stranded mRNA library preparation (including a polyA step selecting eukaryotic mRNA) and sequencing on a NovaSeq 6000 S2 2 × 100 bp. Sequencing generated an average of ~ 75 million reads per sample.

To determine the gene expression patterns of coral host and associated Symbiodiniaceae to incrementally increasing temperatures, reads were processed for reference-based transcriptomics as previously described in Buerger et al. [41]. First, samples passing quality control (*n* = 34) were assessed using FastQC [42], and results were summarized with MultiQC [43]. For identifying and separating the holobiont members (i.e. host and Symbiodiniaceae), reads were first mapped to the *P. lutea* reference genome [12] using the RNA-seq aligner STAR with the –outFilterScoreMinOverLread and –outFilterMatchNminOverLread parameters set to 0.55 (version 2.7.9a; [44]). The unmapped reads were then aligned to the *Cladocopium goreaui* genome [45, 46], which was selected as reference genome due to its higher completeness compared to *Cladocopium* C15 [12], the dominant Symbiodiniaceae genus associated with this coral species [47]. The gene counts generated by STAR (reverse strandness) were processed and imported in R [38] for gene expression analyses. Overall, one Symbiodiniaceae and two host samples were identified as outliers using a standardized connectivity test on network analyses and removed from the analysis [48] (Fig. S1). Genes were assigned to euKaryotic Orthologous Groups (KOG), Gene Ontology (GO) terms and Kyoto Encyclopedia of Genes and Genomes (KEGG) pathways through eggNOG-mapper (version 2.1.6; [49]) on the Galaxy Australia platform [50], with query sequences searched against the eggNOG database 5.0.2 [51] using Diamond [52].

The genome-mapped reads were processed using the limma-voom pipeline (version 3.46.0; [53, 54]). First, low-count genes were removed (‘filterByExpr’ function, min.count = 20), and then normalized data (trimmed mean of the M-values method, TMM) were transformed (log-CPM) and analysed using mixed-effect models for identifying differentially expressed genes (DEGs; Benjamini–Hochberg adjusted *p*-value ≤ 0.05). For the identification of DEGs under incremental temperatures, samples under low heat stress (heat T2) were compared to samples under ambient conditions (heat T0, ambient T0, ambient T2), and samples under moderate heat stress (heat T4) were compared to heat T0, ambient T0, ambient T2 and ambient T4 samples. Colony of origin was included in all mixed models as random effect to account for any host genotype-driven variation.

To identify KOG classes that were significantly enriched with up- or down-regulated genes between treatments, KOG enrichment analyses were run using the Mann–Whitney *U*-test (R package ‘KOGMWU’; [55]). Through the KOGMWU function, delta ranks for 23 KOG gene classes were calculated based on log-fold changes (logFC) generated from the pairwise comparisons between treatments (limma-voom analyses described above). Similarities among up- and down-regulated KOG classes were further explored across heat treatments using Pearson correlations. Similarly, GO enrichment analyses were run following the GO_MWU pipeline (https://github.com/z0on/GO_MWU) on logFC to determine which biological processes were differentially regulated under heat stress.

Sparse partial least-squares discriminant analysis (sPLS-DA), a supervised statistical technique selecting the most discriminative features (i.e. genes in our study) for sample classification [56], was performed separately on host and Symbiodiniaceae transcriptomic data using the R ‘mixOmics’ package (v6.14.1; [57]), following variance stabilizing transformation (‘DESeq2’; [58]). The effect of colony of origin was accounted in the sPLS-DA model through a multilevel approach [59]. Tuning of sPLS-DA was performed to select the optimal number of dimensions and the most discriminative genes (per dimension) between treatment conditions at the lowest error rate, using repeated cross validation (5-folds, 100 repeats) and Mahalanobis distance, resulting in the selection of one component for both datasets, with an error rate of 0.42 and 0.23 for host and Symbiodiniaceae, respectively, across the 500 cross-validation runs (the sample plots from the sPLS-DA are illustrated on two dimensions for visualization purposes).

### 16S rRNA gene amplicon analyses

DNA derived from coral and seawater was extracted using the DNeasy PowerSoil Pro Kit (QIAGEN), according to the manufacturer’s protocol, with the cell lysis step performed using the FastPrep-24 5G bead beater (MP Biomedicals). DNA was also extracted from feeds (rotifers and microalgae) added to the tanks, to act as a control and ensure recovered bacterial 16S rRNA gene reads were derived from coral samples. The V4 region of the 16S rRNA gene was amplified using the forward primer 515F [60] and the reverse primer 806rB [61], for targeting both bacteria and archaea taxa, in a 30 cycle PCR using the AmpliTaq Gold 360 Master Mix (Thermo Fisher) under the following conditions: 95 °C for 10 min, 30 cycles of 95 °C for 30 s, 56 °C for 1 min and 72 °C for 30 s, followed by a final elongation at 72 °C for 7 min. PCR products were sent to the Ramaciotti Centre for Genomics (UNSW, Australia) for standard Illumina library preparation and sequencing on the Illumina MiSeq 2 × 250 bp platform.

Sequence data was analysed in QIIME2 (v 2020.8; [62]). Following removal of poor quality reads and chimeras, amplicon sequence variants (ASVs) were identified based on 100% sequence similarity using DADA2 [63]. Taxonomy was assigned through a Naïve Bayes classifier trained with the feature-classifier plugin using the primers 515F/806rB on the SILVA 132 database [64]. Phylogenetic relationships were built using FastTree [65]. Microbial community analyses were run in R [38], where reads assigned to chloroplasts, eukaryotes and mitochondria were removed as well as contaminants identified using stringent thresholds in the R package ‘decontam’ ([66], P threshold = 0.5; *n* = 1 contaminant identified, Table S1). Singletons and samples with < 5400 reads (*n* = 4) were also excluded from the analyses.

Shannon diversity index was measured on the dataset rarefied to 5400 sequences (‘phyloseq’ package; [67]. Differences in alpha diversity among treatment and time were tested using a linear mixed model (‘glmmTMB’ package; [37]) with colony of origin as fixed effect and tank as random effect; model assumptions were checked through DHARMa residual diagnostics [39]. For beta-diversity analyses, Bray–Curtis dissimilarities were applied to non-rarefied data normalized using proportions and square-root transformation [68], excluding ASVs with an overall relative abundance < 0.001% (9800 ASVs). To determine differences in microbial composition across time (baseline, T0–T5), treatment (ambient, heat) and samples types (coral, seawater, feed), nonmetric multidimensional scaling (NMDS) were generated using ‘phyloseq’ [67], and permutation multivariate analysis of variance (adonis) was run in ‘vegan’ (10,000 permutations; [69]). Dispersion was checked using multivariate homogeneity of group dispersions (‘vegan’ package; [69]), and pairwise comparisons were calculated using Benjamini–Hochberg correction in RVAideMemoire [70]. Differential abundance analyses (‘DESeq2’ package; [58]) were run on filtered data using *p*-value of 0.01, and only significant ASVs present across at least 50% of the samples in one of the compared groups were identified as differentially abundant between groups [71].

### Multilevel integration of omics data

Microbial community composition, Symbiodiniaceae gene expression and host gene expression data were integrated using DIABLO (Data Integration Analysis for Biomarker discovery using Latent components) in ‘mixOmics’ [57], following data normalization (microbiome: centered log-ratio transformation; transcriptomics: normalized variance stabilized counts). DIABLO is a supervised approach that maximizes the common or correlated features across omics datasets retrieved from the same biological samples, to identify key molecular signatures that discriminate between categorical outcomes (i.e. heat treatments in this study) [72]. A multilevel approach was applied to account for the effect of colony of origin; microbial taxa and annotated genes (KEGG orthology) were used as input for these analyses, which corresponded to 3916 ASVs, 7627 genes for the Symbiodiniaceae dataset and 12,435 for the host. A design matrix of 0.6 was selected (i.e. prioritizing correlation over discrimination) and applied using 50 × fivefold cross-validation (Mahalanobis distance), to identify the optimal number of component and variables.

## Results

### Holobiont performance declines from 1 DHW

**Fig. 2:**
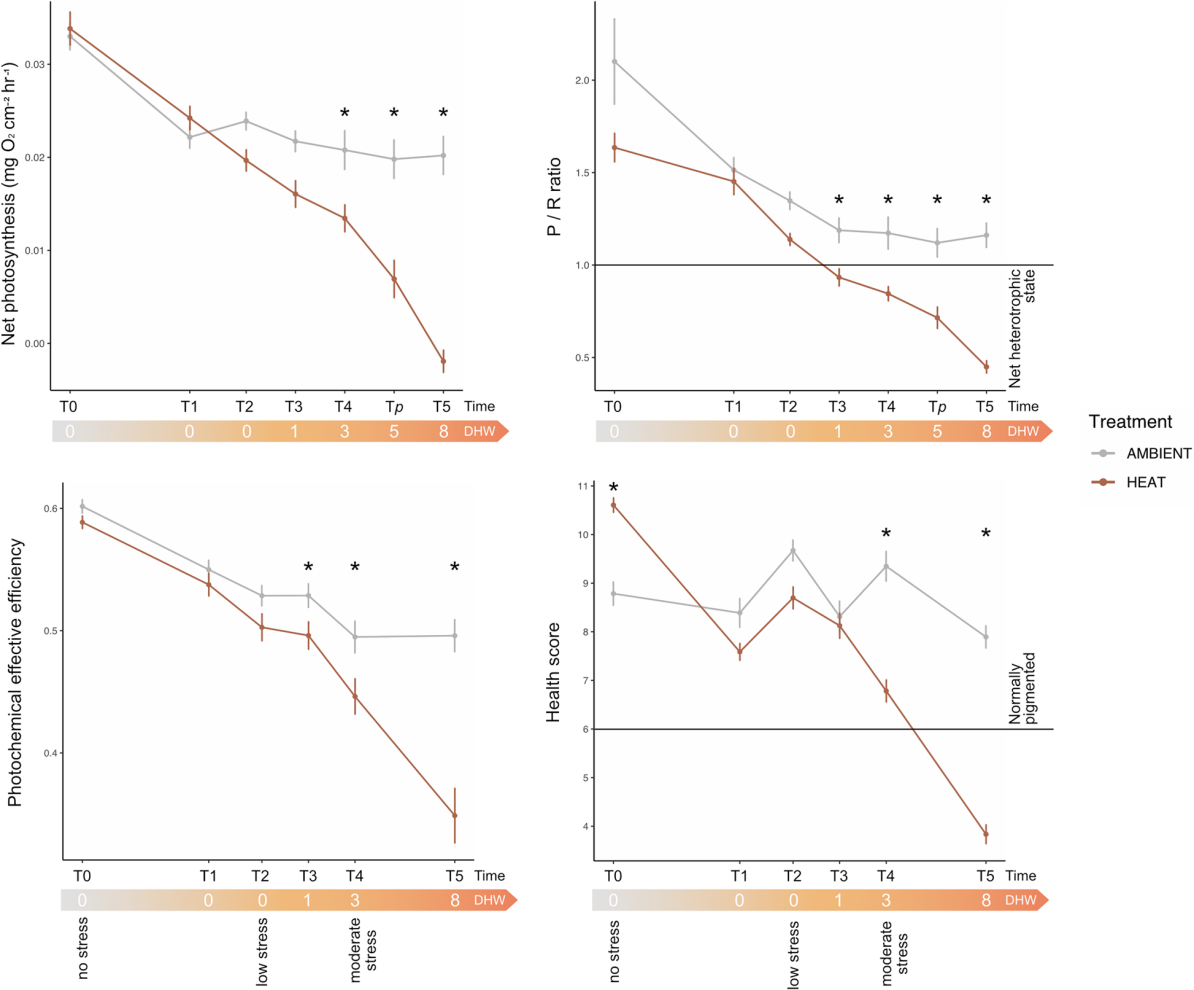
Holobiont physiological health metrics under increasing heat stress. Net photosynthesis, photosynthesis/respiration ratio (P/R ratio; P/R < 1 indicates a net heterotrophic state), photochemical effective efficiency (∆F/Fm′) and health score (health score ≥ 6 indicates normally pigmented corals) are shown over time (T0–T5) under increasing temperatures; DHW are reported for the heat treatment only (DHW = 0 under ambient conditions). Net photosynthesis and P/R ratio were measured at an additional time point (T*p*) corresponding to 5 DHW. Mean ± SE is shown over time, with asterisks indicating significantly different physiological responses between ambient and heat treatment at each time point (*p* < 0.05, adjusted post hoc tests)

Corals exposed to increasing temperatures showed signs of reduced physiological performance at 1 DHW. Photosynthesis rates gradually declined with increasing temperatures, and significant differences between treatments were observed from 3 DHW (Fig. 2, Table S2). In contrast, R rates showed a (albeit insignificant) tendency to increase with elevated temperatures (Fig. S2, Table S2). Consequently, P/R ratio significantly declined with increasing temperature from 1 DHW onwards with ratios dropping below the autotrophic compensation point of 1 (Fig. 2, Table S2). This coincided with a significant reduction in the effective quantum yield of coral holobionts (Fig. 2, Table S2). Ultimately, the reduced photophysiological performance of coral holobionts was also reflected in their health scores (pigmentation). A significant decline was observed from 3 DHW, with corals under 3 and 8 DHW showing an average decrease of 2.6 and 4.1 units respectively (Fig. 2 and Table S2). Despite the significant loss in pigmentation compared to ambient conditions, corals under 3 DHW were characterized by an average health score above 6 (health score 6.8 ± 1.1; [31]), indicating corals were still healthy, whereas exposure to higher heat stress resulted in coral bleaching (8 DHW, health score 3.8 ± 0.7). Finally, it is important to note that the physiological health metrics initially declined under both ambient and heat treatments (Fig. 2). However, initial significant differences were identified solely in the health scores, which remained within the values observed in normally pigmented corals (Fig. 2).

### Host transcriptional responses to incremental heat stress

**Fig. 3:**
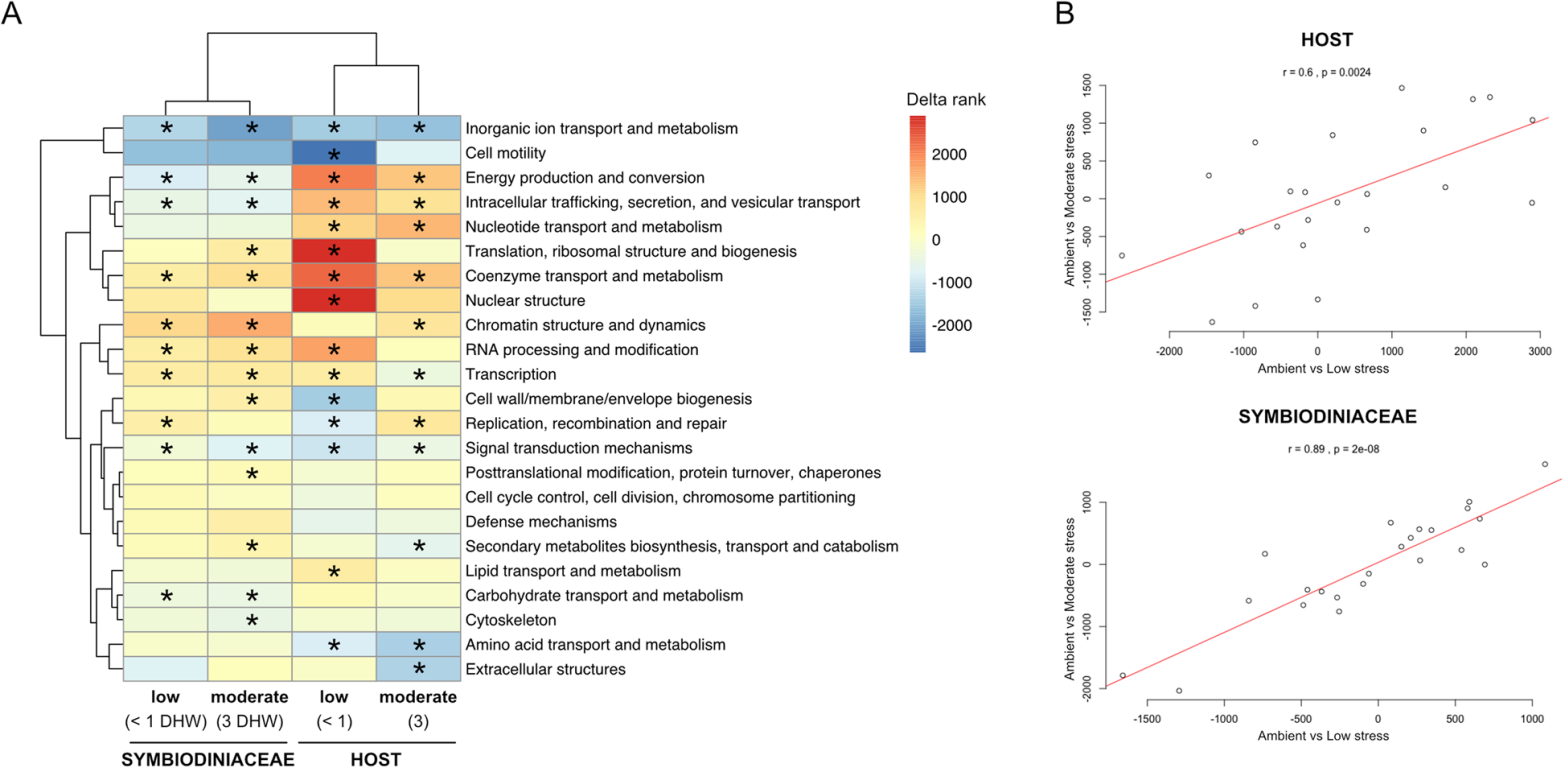
EuKaryotic Orthologous Group (KOG) enrichment analysis in host and Symbiodiniaceae during early heat stress. **A** Heat map illustrating KOG categories up- (red) and down- (blue) regulated in low (< 1 DHW; T2) and moderate (3 DHW; T4) heat stress compared to ambient conditions in Symbiodiniaceae and host. Asterisks indicate KOG categories significantly enriched with up- or down-regulated genes. **B** Correlation between KOG delta ranks between low stress vs ambient, and moderate stress vs ambient in host and Symbiodiniaceae

An average of 47% of host-derived transcriptional reads mapped to the reference (*P. lutea* genome, Table S3), representing an average of 35.5 million reads per sample. Despite a strong genotype effect, with colony of origin explaining most of the total variation in gene expression patterns (Fig. S3), expression profiles were also affected by heat stress. Compared to ambient conditions, 15 and 12 KOGs were enriched under low (< 1 DHW; T2) and under moderate (3 DHW; T4) stress, respectively, with overall significant correlation between low and moderate stress enrichment patterns (*r* = 0.6, *p* < 0.01; Fig. 3). Among these patterns, the expression of genes related to ‘energy production’ was upregulated under heat stress, while processes related to ‘inorganic ion and amino acid transport and metabolism’ were downregulated. Furthermore, genes related to ‘replication, recombination and repair’ were significantly downregulated under low stress while upregulated under moderate stress, while genes associated with ‘translation, ribosomal structure and biogenesis’ were significantly upregulated under low stress only (Fig. 3A).

These overall expression patterns were corroborated by the GO enrichment analysis. Heat stress caused a significant upregulation of genes involved in processes related to the tricarboxylic acid (TCA) cycle, as well as adenosine triphosphate (ATP), lipid and glucose metabolism, and protein catabolism (Tables S4 and S5). At the same time, apoptotic pathways were positively regulated under both low and moderate stress (Tables S4 and S5). In addition to these universal heat stress responses, there were also differences in the GO enrichment patterns between low and moderate heat stress. While anion transport was positively regulated under low stress conditions, it was downregulated under moderate stress conditions, together with bicarbonate transport (Tables S4 and S5).

**Fig. 4:**
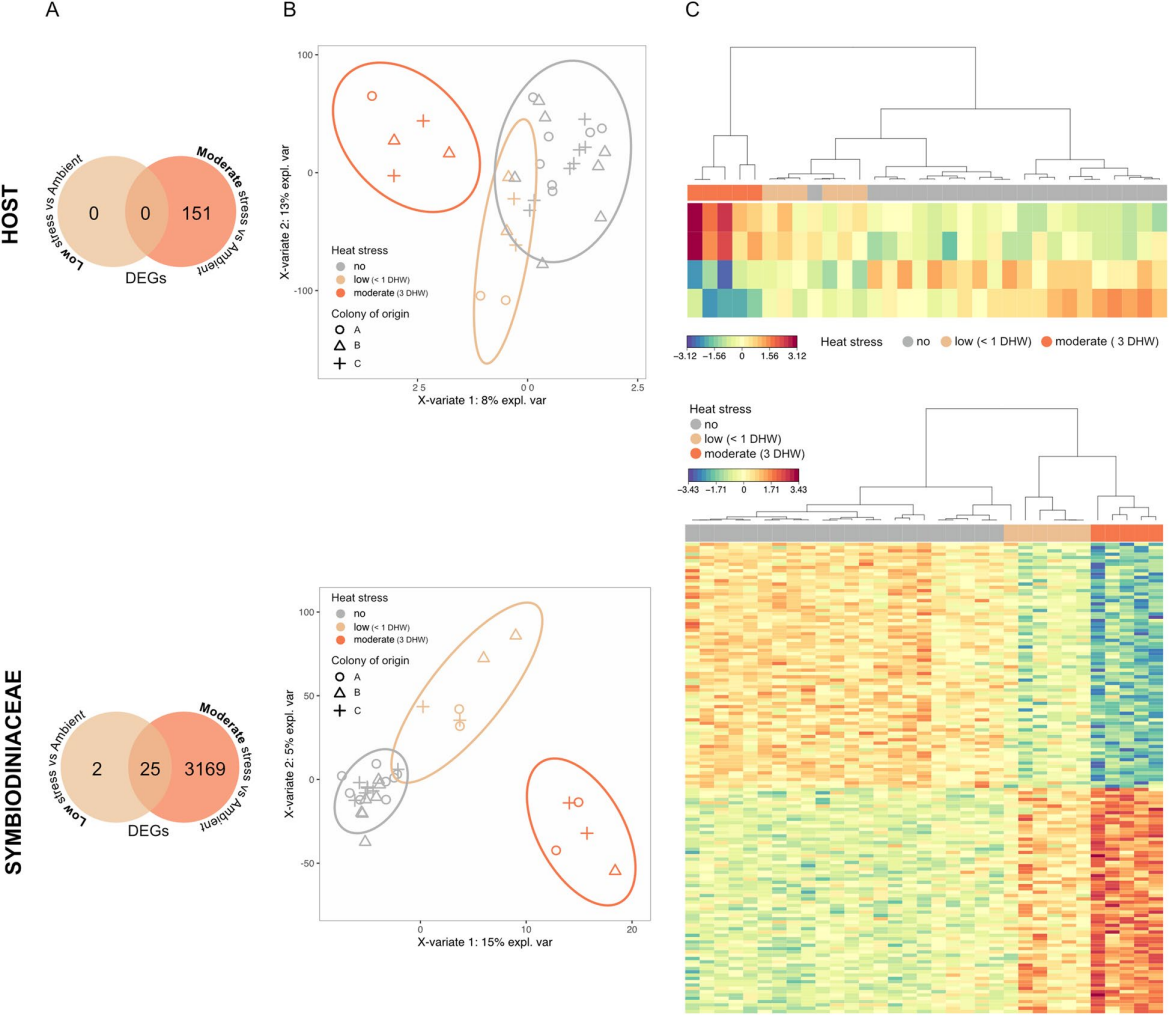
Gene expression profiles of coral host and Symbiodiniaceae during early heat stress. **A** Venn diagram of the number of differentially expressed genes (DEGs) in corals exposed to low (< 1 DHW; T2) or moderate (3 DHW; T4) stress compared to ambient conditions determined by voom-limma analyses. **B** Multilevel sPLS-DA for host and Symbiodiniaceae gene profiles; samples between treatment conditions are discriminated along component 1. **C** Heat map illustrating the top discriminative genes between ambient, low, and moderate stress, identified along with component 1 of multilevel sPLS-DA analysis for host (*n* = 4 genes) and Symbiodiniaceae (*n* = 142 genes)

Differential expression analyses identified 151 genes differentially expressed under moderate stress compared to ambient conditions (total of 24,207 genes; Fig. 4A and Table S6), while no DEGs were detected at low stress. Among the identified DEGs under moderate stress, genes encoding for glutamate synthase (GOGAT) and phosphoserine aminotransferase (PSAT), which are key genes in amino acids biosynthetic processes, were significantly downregulated (Table S6). Additional genes associated with the de novo serine biosynthesis pathway [9] were also downregulated (albeit not significantly) under low and moderate stress, while multiple genes involved in the glycine/serine biosynthesis pathway dependent on food-derived choline [9] showed an increase in expression under these conditions (i.e. dimethylglycine dehydrogenase [DMGDH], sarcosine dehydrogenase [SARDH]; Table S7). In addition, four discriminative genes were selected through multilevel sPLS-DA analysis (Fig. 4B and C, Table S8).

### Symbiodiniaceae transcriptional responses to incremental heat stress

On average, 11.1 million reads per sample uniquely mapped to the *Cladocopium* reference genome (Table S3), representing the gene expression of host-associated Symbiodiniaceae. Similar to host gene expression analyses, colony of origin had a strong effect on Symbiodiniaceae transcriptional responses, representing the largest source of variation (Fig. S3). However, transcriptional changes were also detected according to heat stress conditions. At a broad functional level, KOG enrichment analyses revealed that genes related to 10 functional classes were differentially regulated under low stress and 14 classes under moderate stress (Fig. 3A). Genes related to ‘replication, recombination and repair’ were the most upregulated under low stress and ‘RNA processing and modification’ under moderate stress (adjusted *p*-values), while genes related to ‘inorganic ion and carbohydrate transport and metabolism’, and ‘energy production and conservation’, were consistently downregulated under both low and moderate stress. Importantly, enrichment patterns observed under low stress were similar to moderate stress, with some additional KOG classes significantly enriched under moderate stress only, such as ‘posttranslational modification, protein turnover, chaperones’ and ‘cytoskeleton’ (Fig. 3A). This incremental response to stress intensity was confirmed by a high correlation of KOG delta ranks for low stress vs ambient to moderate stress vs ambient (*r* = 0.89, *p* < 0.01; Fig. 3B); this relationship was stronger than in the host.

GO enrichment analyses further characterized the differences in expression among functional classes. Genes encoding for inorganic ion transmembrane transport, in particular for ammonium transmembrane transport, were significantly downregulated under low stress in Symbiodiniaceae (Table S9). This expression profile was intensified under moderate stress, in conjunction with the upregulation of more general heat stress responses such as transcription regulation, histone modification, protein folding and lipid membrane responses (Table S10).

When comparing Symbiodiniaceae transcription profiles under heat stress to ambient conditions at the gene level, differential expression analyses identified a total of 27 genes differentially expressed under low stress and 3194 genes under moderate stress (total of 23,345 genes; Fig. 4A, Tables S11 and S12). Among the latter, genes related to ammonium transport, glutamine synthetase and glutamate synthase were consistently downregulated, while genes related to positive TORC1 signalling were upregulated (Table S12). Furthermore, multilevel sPLS-DA identified 142 discriminative genes between heat stress and ambient conditions (Fig. 4B and C), including glutamine synthetase (Table S13). In comparison to the host, sPLS-DA analyses on the Symbiodiniaceae genes showed a higher accuracy in discriminating between experimental treatments (77% compared to 58% accuracy).

### Microbial community shifts from 1 DHW

**Fig. 5:**
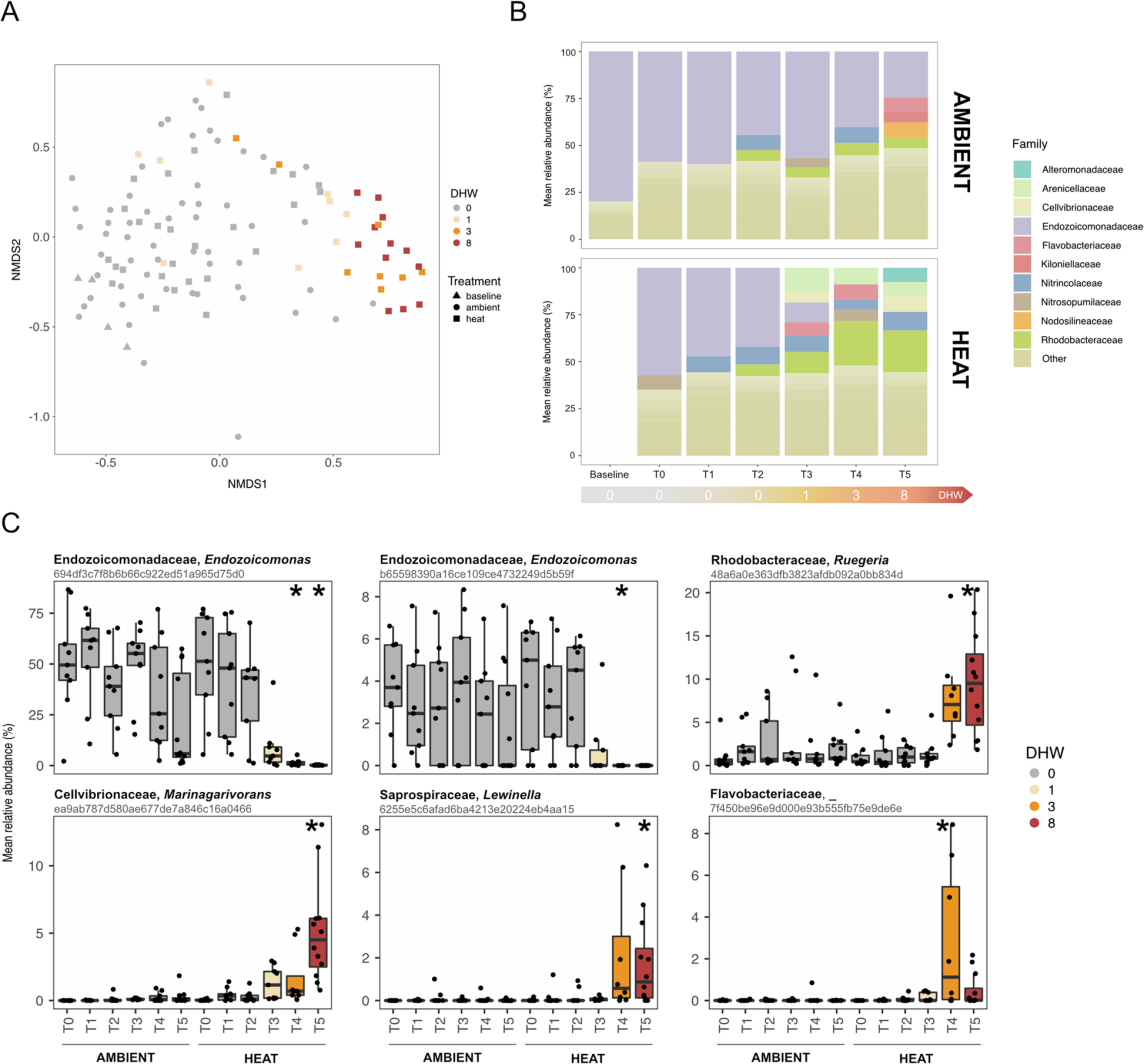
Coral microbial responses to increasing heat stress determined by 16S rRNA gene sequencing. **A** Non-metric multidimensional scaling (NMDS, sqrt-rooted data; stress = 0.21) based on Bray–Curtis dissimilarities calculated on relative abundance of ASVs present in the coral samples preceding fragging (baseline) and during the heat stress experiment (ambient and heat treatments). **B** Mean relative abundance of prevalent microbial families (> 5% relative abundance) in the coral samples under ambient and heat conditions across time (baseline, T0–T5). DHW are reported for the heat treatment only (DHW = 0 under ambient conditions). **C** Top differentially abundant ASVs between ambient and heat treatment identified using DESeq analyses (*p* < 0.01, adjusted post hoc tests). The relative abundance of the ASVs is shown over time in each treatment. Significantly different relative abundance of a specific ASV under heat treatment compared to ambient conditions (for the respective time point) are indicated with an asterisk; the taxonomic assignment for each ASV is shown as family and genus. Box, interquartile range (IQR); line in box, median; whiskers, minimum and maximum values not outliers (i.e. − / + 1.5 × IQR)

A total of 2,723,455 high-quality 16S rRNA amplicon reads, with an average of 19,315 reads per sample (5411 min, 84,479 max; blanks excluded), were retrieved with 12,558 amplicon sequence variants (ASVs) identified after quality trimming, chimera removal and data filtration. Rarefaction curves plateaued indicating that sequencing depth was sufficient to assess diversity of the coral associated microbes (Fig. S4). Although homogeneity of dispersion was not met, microbial communities associated with the coral tissue were clearly distinct from the microbiomes of the seawater and feeds (rotifers and microalgae; Figs. S5 and S6, Table S14). When exposed to incremental temperatures, the coral microbial community remained stable until cumulative thermal stress reached 1 DHW, after which significant differences in community structure were observed for the 1, 3 and 8 DHW samples when directly compared to the respective time point under ambient conditions (Fig. 5 and Table S15). Specifically, Rhodobacteraceae (*Alphaproteobacteria*)-affiliated sequences increased from 6% (ambient) to 21% under 8 DHW (Figs. 5B and S7). In contrast, the dominant microbial family under ambient conditions, Endozoicomonadaceae (*Gammaproteobacteria*), represented on average 57% of the community at the ambient T0 time point but accounted for only 0.2% of retrieved sequences when thermal stress exposure reached 8 DHW (Figs. 5B and S7).

When exploring microbial changes at the ASV level, we identified 1, 3 and 15 differentially abundant ASVs between ambient and 1, 3 and 8 DHW, respectively (present in at least 50% of the samples within each category; Figs. 5C and S8). Among these ASVs, two members of the family Endozoicomonadaceae were consistently reduced in relative abundance at 3 and 8 DHW (Fig. 5C), while ASVs affiliated to Rhodobacteraceae and Flavobacteriaceae, for example, were significantly increased under heat stress (Figs. 5 and S8). Importantly, the coral microbial community was influenced by time in addition to heat stress, with a distinct community structure at T5 compared to T0 under ambient conditions (Fig. 5B and Table S15). An effect of the colony of origin was also detected, with microbial structure being significantly different among the three colonies (Table S15). Despite these differences, samples within each colony clustered at ≥ 3 DHW (Fig. S9), suggesting similar responses among colonies to the increasing thermal stress. Alpha diversity (Shannon diversity index) differed between colonies, but did not vary significantly across time and treatment with the exception of an overall higher diversity at T5 compared to the other time points (Fig. S10, Table S16).

### Integrated responses to heat stress

**Fig. 6:**
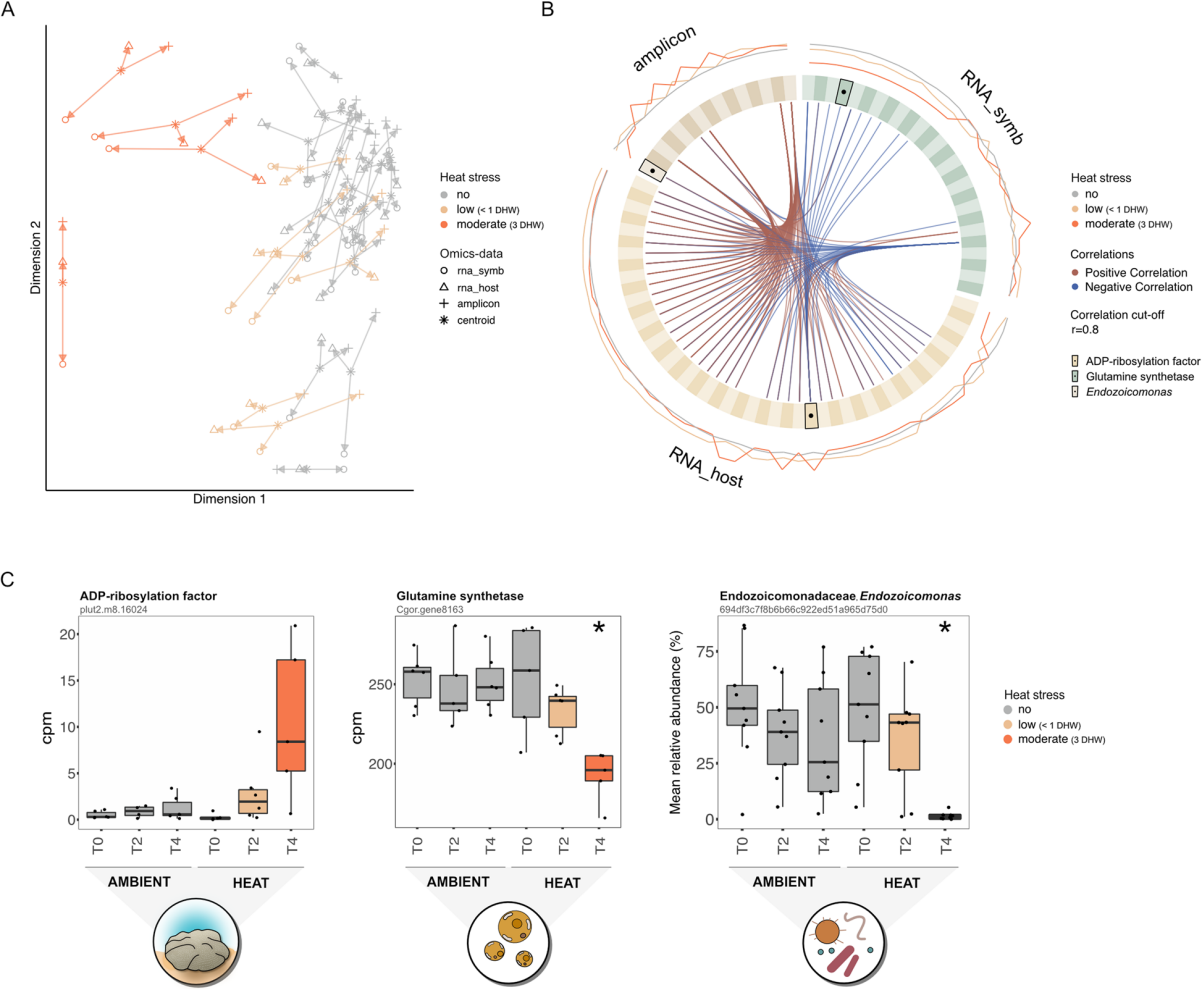
Integrative analysis of host (transcriptomics, annotated genes only), Symbiodiniaceae (transcriptomics, annotated genes only) and microbial (amplicon) profiles in coral samples under ambient (‘no stress’), low (< 1 DHW) and moderate (3 DHW) stress conditions using DIABLO. **A** Arrow plot showing the agreement between the different omic datasets at the sample level, with * indicating the centroid between the datasets for each sample under treatment conditions shown by colour. Short arrows indicate strong agreement between datasets. **B** Circos plot visualizing positive and negative correlations (*r* > 0.8, represented as red and blue lines) between the most discriminative features of each omics dataset along DIABLO components 1 and 2 (see Fig. S11 for details). Each quadrant represents an omics dataset: microbial taxa (‘amplicon’, light and dark brown), host genes (‘rna_host’, light and dark yellow) and Symbiodiniaceae genes (‘rna-symb’, light and dark green); ADP-ribosylation factor (host gene), glutamine synthetase (Symbiodiniaceae gene) and *Endozoicomonas* (microbial taxa) are highlighted on the plot by black outlines with a central dot. The most outer lines represent the abundance level of each variable under ambient, low stress and moderate stress. **C** Based on DIABLO analyses, the abundance of the host gene encoding for ADP-ribosylation factor was negatively correlated to the Symbiodiniaceae gene encoding for glutamine synthetase, as well as the relative abundance of *Endozoicomonas* bacteria; abundance levels for each feature are shown here in counts per million (CPM; genes) and mean relative abundance (ASV)

To link the heat-stress responses among holobiont members, we integrated the three omics datasets (i.e. microbiome, gene expression of Symbiodiniaceae and host) through DIABLO. These analyses confirmed that samples under moderate stress were distinct from ambient conditions across datasets (Fig. 6A). However, there was similarity between samples under low stress and ambient conditions for host gene expression and particularly for microbial structure (Fig. 6A). When exploring associations between features from the three datasets, we identified a total of 88 highly correlated variables (i.e. 47 host genes, 26 Symbiodiniaceae genes and 15 ASVs) along the first two principal components (Fig. 6B, Fig. S11, Table S17). Importantly, a host ADP-ribosylation factor (plut2.m8.16024) that was most expressed under moderate stress (Fig. 6B and C) correlated with 11 Symbiodiniaceae genes (including a negative correlation with symbiont glutamine synthetase, Fig. 6B and C) and two ASVs (including a negative correlation with an *Endozoicomonas* ASVs, Fig. 6B and C). Among the Symbiodiaceae genes, a nitrite transporter (Cgor.gene11242) that was most expressed under moderate stress correlated with 28 host genes (largely dominated by negative correlations with genes involved in transcriptional and translational regulation) and 1 Arenicellaceae ASV (Fig. S11). Lastly, the relative abundance of four ASVs (assigned to Cellvibrionaceae, Rhodobacteraceae, Sandaracinaceae and Arenicellaceae families) was positively correlated with a wide range of host genes, but no strong correlations were detected with any Symbiodiniaceae genes, with the exception of one Arenicellaceae ASV that was negatively correlated to one Symbiodiniaceae nitrite transporter (Fig. S11).

## Discussion

The stability of the coral holobiont under heat stress depends on the interactions between all its members (host, Symbiodiniaceae and microbes) [4]. In a stable state, mutualistic nutrient recycling within the holobiont underpins its ecological success in oligotrophic waters. Here, our integrative analysis within a thermotolerant coral reveals that even low levels of heat stress (i.e. < 1 DHW) may undermine this ecological foundation. Reduced photosynthesis and enhanced host metabolic energy demand drive the coral holobiont to a net heterotrophic state (1 DHW) in which algal-derived photosynthates are insufficient to cover its metabolic requirements. The destabilization of this association is linked to a shift of gene expression profiles towards an aposymbiotic-resembling state and a strong restructuring of the microbial community.

The host gene expression profiles revealed that this metabolic switch occurred well before any visual signs of symbiosis breakdown (i.e. coral bleaching). Even at low heat stress (< 1 DHW), genes related to cellular energy production, ATP, lipid and carbohydrate metabolism were significantly enriched, indicating elevated carbon turnover and consumption in the host cells. This aligns with previous studies suggesting that heat stress can shift the host metabolism from a nitrogen to a carbon-limited state [6, 73]. The reduced availability of algal-derived carbon may, in turn, alter host amino acid metabolism, promoting a net release of ammonium [6]. The increased protein catabolism observed in our study suggests that even early stages of heat stress may reduce the host ability of maintaining a nitrogen-limited state, which is prerequisite of a stable symbiosis [22, 23].

The transcriptional changes observed in the host were strictly linked to the Symbiodiniaceae gene expression profile. A significant downregulation of genes involved in ammonium transport and fixation was observed from low heat stress levels (Tables S9 and S10), reflecting the potential increased nitrogen availability for the algal symbionts under these conditions, which occurred prior to the observed thermal-induced decline in holobiont health. Our findings suggest that this decoupling of carbon recycling and the loss of a nitrogen-limited state in the symbiosis gradually accelerate with increasing temperatures. In the host, the significant downregulation of key genes in the de novo serine biosynthesis pathway (GOGAT, PSAT) indicates that a reduced availability of algal-derived carbon alters the amino acid metabolism, forcing the host to rely on fixed nitrogen sources (e.g. choline; [9]). Indeed, multiple host genes involved in the glycine/serine biosynthesis via food-derived choline were upregulated (albeit not significantly) under moderate stress. In this context, the downregulation of ammonium and anion transporters coupled with the upregulation of Symbiodiniaceae genes involved in positive TORC1 signalling supports the idea that enhanced inorganic nutrient availability may stimulate anabolic activity and growth of algal symbionts at early stages of heat stress. However, host genes related to immune responses and the apoptotic clearance of cells were also upregulated under these conditions. Similarly, Symbiodiniaceae showed an upregulation of multiple biological processes commonly related to heat stress, including transcription regulation, histone modification, protein folding and lipid membrane responses, suggesting host and Symbiodiniaceae cell homeostasis were impaired at 3 DHW. Consistent with this, holobiont health metrics showed a significant decline in photosynthetic performance and effective photochemical efficiency. Hence, our results indicate that the destabilization of nutrient cycling in the coral-Symbiodiniaceae precedes the onset of the processes ultimately leading to the complete breakdown of the symbiosis (i.e. bleaching).

Based on the output of the multilevel sPLS-DA analysis and the number of DEGs between heat stress and ambient conditions, Symbiodiniaceae showed a stronger response than the host, potentially suggesting Symbiodiniaceae may be the first responder to environmental changes. These results contrast with previous studies that identified the host as more transcriptionally responsive to high temperatures [4, 14, 74–78]. However, it is possible this variance is linked to distinct mechanisms underpinning stress responses between thermotolerant (this study) and environmentally sensitive coral species [74, 75, 77, 78]. Another factor likely contributing to these contrasting results is stress severity; while our study focuses on early stages of stress, the majority of heat stress studies performed to date have explored transcriptomic profiles under higher stress intensity [4, 13, 14, 76]. In addition, methodological differences (reference-based vs de novo transcriptomics) may have also influenced the number of DEGs detected under heat stress across studies. On this note, Symbiodiniaceae differential expression analysis in our study may not be truly reflective of changes occurring at the community level.

The effects of heat stress within the coral holobiont were not restricted to the destabilization of the coral-Symbiodiniaceae symbiosis. Coral exposure to incremental temperatures resulted in a significant shift in the microbial community structure from as early as 1 DHW (i.e. coinciding with a metabolic shift within the coral holobiont towards a net heterotrophic state), with shifts becoming increasingly pronounced through to 8 DHW. The significant reduction of two *Endozoicomonas* ASVs under heat stress indicates the restructuring of the microbial community was likely related to dysbiosis. Indeed, these bacteria commonly dominate the microbiome of healthy corals and show high degrees of host specificity and co-phylogeny [79–81]. While the specific contribution of *Endozoicomonas* to coral holobiont functioning is not yet fully understood, these bacteria are considered beneficial for coral health due to their putative role in carbon, phosphate and sulphur cycles, as well as amino acids and vitamin biosynthesis [12, 82–84]. Concurrent with a decrease in *Endozoicomonas*-affiliated sequences, we observed an increase in the relative abundance of opportunistic bacteria, such as Rhodobacteraceae. However, some microbial taxa within this family have been proposed as beneficial under heat stress, with some *Ruegeria* members being a notable example [24, 85]. Nevertheless, similar microbial shifts have been previously reported across a wide range of coral taxa exposed to environmental stress [14, 78, 86], indicating these microbial responses are commonly associated with general coral stress responses. Hence, the shifts in microbial composition observed in this study are unlikely an adaptive response; instead, they may exacerbate the negative impacts of heat stress on other holobiont members. Microbial functional potential should be further investigated to gain a comprehensive understanding of the effects of the identified microbial community changes on coral holobiont health.

We identified strong correlations between the responses of all coral holobiont members. Among these correlations, the upregulation of one host gene (an ADP-ribosylation factor) during heat stress coincided with the downregulation of critical genes involved in algal nitrogen metabolism (glutamine synthetase) and the decline in *Endozoicomonas*-affiliated sequences. ADP-ribosylation factors regulate host intracellular vesicle transport and have been proposed to be key negative regulators in cnidarian-Symbiodiniaceae symbioses [87]. In support of this hypothesis, our results suggest that the upregulation may not only negatively affect host-associated Symbiodiniaceae populations but may also influence the regulation of the dominant bacterial symbiont, *Endozoicomonas*. Previous studies showed that *Endozoicomonas* occupy an endosymbiotic niche, forming aggregates termed coral-associated microbial aggregates (CAMAs) within the coral holobiont tissues [79, 84, 88] and could thus be subjected to the same host regulatory machinery. Furthermore, the regulation of genes involved in symbiosis networks have been suggested to be critical in the stability of cnidarian symbiotic relationships under heat stress, with symbiosis-related genes responding prior to any visible signs of symbiosis breakdown [89]. Experimental validation is required to clarify the link between the observed correlations and thermal stress responses.

## Conclusions

**Fig. 7:**
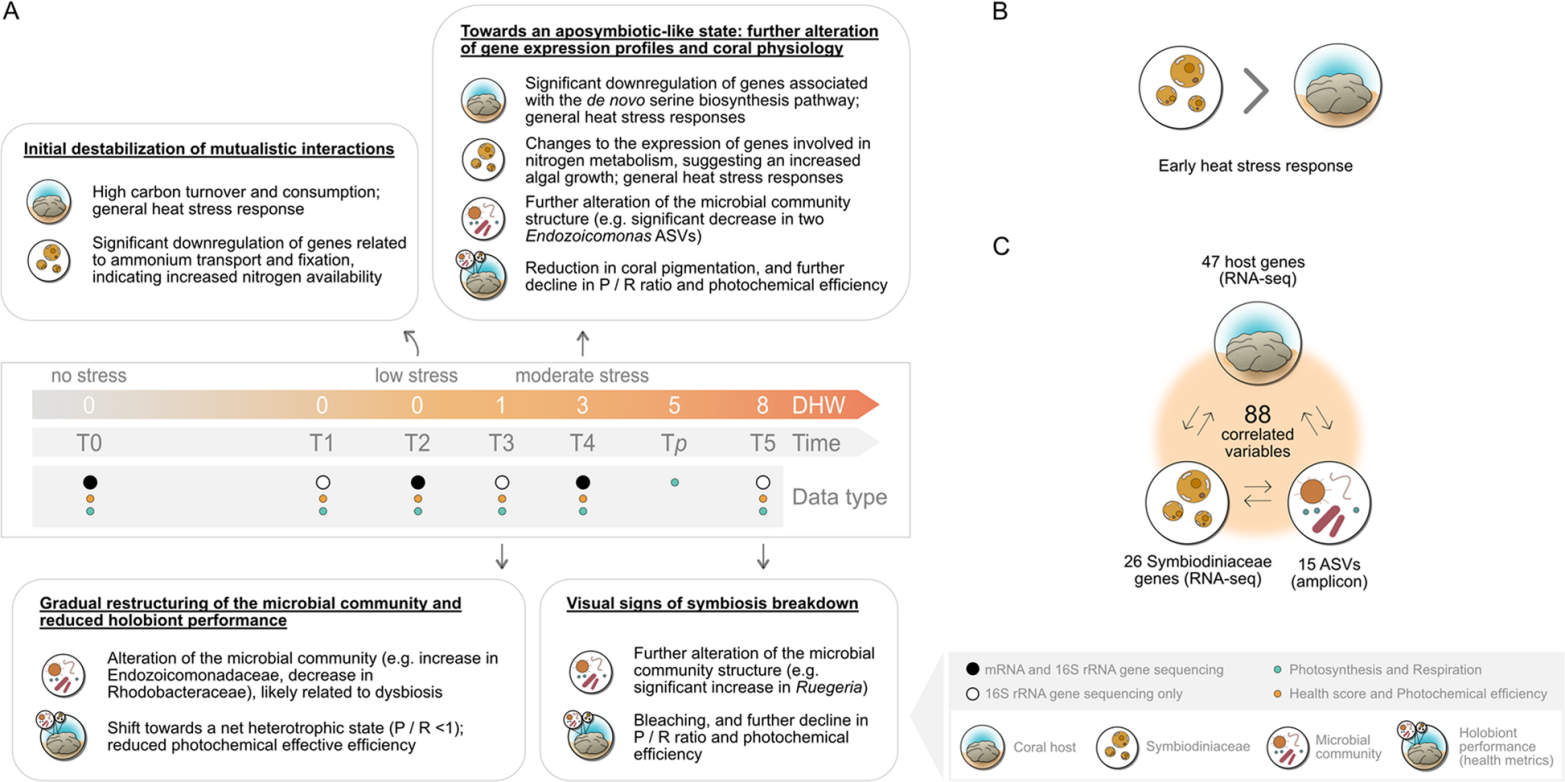
Schematic summary of the coral holobiont responses to increasing temperatures. **A** Temporal responses of the coral host (RNA-seq), Symbiodiniaceae (RNA-seq) and microbial community (amplicon) to increasing heat stress (degree heating weeks, DHW) in the coral *P. lutea* and the associated holobiont performance (physiological health metrics). **B** Symbiodiniaceae showed a more pronounced response to heat stress than the host, based on number of differentially expressed genes (DEGs) and multilevel sPLS-DA analysis. **C** Integrative multi-omics analyses identified key correlations between the responses of holobiont members under heat stress

Our results show that the breakdown of the symbiosis under heat stress is not driven by one coral holobiont member in isolation (summarized in Fig. 7). Despite the limited replication in individual coral colonies (*n* = 3), our physiological and transcriptome data suggest that changes in nutrient cycling and availability begin under early stages of heat stress. Increased metabolic turnover and catabolic production of inorganic nutrients gradually shift the holobiont from a nitrogen- to a carbon-limited state. Under these conditions, Symbiodiniaceae may initially benefit from the enhanced nutrient availability but, in turn, may reduce the translocation of fixed carbon to the host, destabilizing their mutualistic interaction. In addition, our results reveal that the altered symbiotic and nutritional state within the holobiont may also drive changes in the associated microbial community, with mutualistic symbionts being gradually replaced by more opportunistic microbes. Hence, a gradual destabilization of mutualistic interactions within the coral holobiont commences at low heat stress levels (< 1 DHW). This destabilization ultimately leads to coral physiology and gene expression profiles increasingly resembling a cnidarian aposymbiotic-like state, with a reduced contribution of symbionts to host metabolism and functioning. Our study highlights the importance of applying integrative multi-omics approaches for predicting coral reef functioning under future climate.

## Supplementary Information

Supplementary Material 1. Supplementary figures: Fig S1. Sample clustering for outlier detection in host and Symbiodiniaceae. Heat stress (no; low: < 1 DHW; moderate: 3 DHW) is defined here as ‘stress’. Only samples passing QC are included. Fig S2. Coral respiration rates throughout the heat stress experiment. Mean ± SE is shown over time, and DHW are reported for the heat treatment only (DHW = 0 under ambient conditions). Fig S3. Principal Component Analysis (PCA) of gene expression profiles in host and Symbiodiniaceae on transformed (variance stabilizing transformation) and centered data (left) and multilevel PCA on the same dataset (right) to account for the effect of colony of origin. Fig S4. ASV rarefaction curves of 16S rRNA gene sequences for coral (baseline, T0-T5), seawater and feed (rotifers and microalgae) samples. Fig S5. Non-metric multi-dimensional scaling (NMDS, sqrt-rooted data; stress = 0.17) based on Bray–Curtis dissimilarities calculated on relative abundance of ASVs in coral (*Porites lutea*), seawater and feed (rotifers and microalgae) samples. Fig S6. Mean relative abundance of dominant microbial families in seawater and feed (rotifers and microalgae) samples. For seawater, the microbial community structure is shown under ambient and heat treatment over time (T0, T3, T5). Fig S7. Changes in relative abundance of the dominant 10 microbial families (> 5% mean relative abundance) across treatments (ambient, heat) and time (baseline, T0—T5) in the coral samples throughout the heat stress experiment. Box = inter-quartile range (IQR), line in box = median, whiskers = minimum and maximum values not outliers (i.e. -/ + 1.5*IQR). Fig S8. Changes in mean relative abundance of differentially abundant ASVs between ambient and heat treatment over time (T0-T5). Differentially abundant ASVs were identified using DESeq (*p* < 0.01; adjusted post hoc tests), with the restriction that only ASVs present in at least 50% of the samples in one of the compared groups were considered. Significant differences are indicated with asterisks (heat vs ambient for the respective time point). Taxonomic assignment for each ASV is shown as Family, Genus, Species. Box = inter-quartile range (IQR), line in box = median, whiskers = minimum and maximum values not outliers (i.e. -/ + 1.5*IQR). Fig S9. NMDS (sqrt-rooted data; stress = 0.21) based on Bray–Curtis dissimilarities calculated on relative abundance of ASVs in coral samples during the heat stress experiment (baseline, ambient, heat) in the three colonies of origin (A, B, C). Fig S10. Shannon diversity index representing alpha diversity of the microbiome in the coral samples over time (baseline, T0-T5) and across treatments (ambient and heat). Box = inter-quartile range (IQR), line in box = median, whiskers = minimum and maximum values not outliers (i.e. -/ + 1.5*IQR). Fig S11. The most discriminative features of each omics-dataset along component 1 and 2 (based on DIABLO analyses), and their correlations. The most outer lines represent the abundance level of each variable under ambient, low stress and moderate stress. Features’ taxonomy / functional annotations are outlined in Table S17.

Supplementary Material 2. Supplementary tables: Table S1. One ASV was identified as contaminant using the R package decontam (https://github.com/benjjneb/decontam). Table S2. Generalized linear mixed models tested the fixed effect of treatment (ambient, heat), time (T0, T1, T2, T3, T4, T*p*, T5), colony of origin (A, B, C) and the interaction between treatment and time on: health score, photosynthesis (P; sqrt-transformed), respiration (R; absolute), Gross P:R and photochemical effective efficiency (sqrt-transformed) in the coral *Porites lutea.* Tank and fragment were included in the model to account for repeated measures. The pairwise comparisons (Šidàk correction) for significant interactions are reported here. Estimates are not back-transformed. Significant factors (*p* < 0.05) are indicated in bold. Table S3. (separate Dataset file). Summary of sequencing statistics of the transcriptomic dataset, and percentage of reads mapping to the reference genomes. Table S4. (separate Dataset file). Outcome of the Gene Ontology (GO) enrichment analyses (Biological Processes) for the coral host between low stress (< 1 DHW) and ambient conditions; only significant genes (adjusted *p* < 0.05) are shown. Table S5. (separate Dataset file). Outcome of the Gene Ontology (GO) enrichment analyses (Biological Processes) for the coral host between moderate stress (3 DHW) and ambient conditions; only significant genes (adjusted *p* < 0.05) are shown. Table S6. (separate Dataset file). Overview of the coral host Differentially Expressed Genes (DEGs) detected between moderate stress (3 DHW) and ambient conditions. Table S7. (separate Dataset file). Overview of the coral host gene expression under moderate stress (3 DHW) compared to ambient conditions, shown as log-fold-changes (logFC). The dataset excludes Differentially Expressed Genes (DEGs), as listed in Table S6. Table S8. (separate Dataset file). Overview of the discriminative coral host genes selected through multilevel sPLS-DA analysis. Table S9. (separate Dataset file). Outcome of the Gene Ontology (GO) enrichment analyses (Biological Processes) for the coral-associated Symbiodiniaceae between low stress (< 1 DHW) and ambient conditions; only significant genes (adjusted *p* < 0.05) are shown. Table S10. (separate Dataset file). Outcome of the Gene Ontology (GO) enrichment analyses (Biological Processes) for the coral-associated Symbiodiniaceae between moderate stress (3 DHW) and ambient conditions; only significant genes (adjusted *p* < 0.05) are shown. Table S11. (separate Dataset file). Overview of Symbiodiniaceae Differentially Expressed Genes (DEGs) detected between low stress (< 1 DHW) and ambient conditions. Table S12. (separate Dataset file). Overview of Symbiodiniaceae Differentially Expressed Genes (DEGs) detected between moderate stress (3 DHW) and ambient conditions. Table S13. (separate Dataset file). Overview of the discriminative Symbiodiniaceae genes selected through multilevel sPLS-DA analysis. Table S14. Permutation multivariate analysis of variance (adonis function in R vegan package) based on Bray–Curtis dissimilarities applied on square-root-transformed relative abundances to examine the effects of sample type (fixed, three levels: “coral”, “seawater”, “feed”) on the microbiome. *P*-values were calculated using 10,000 permutations and statistical significance (*p* < 0.05) is shown in bold. Table S15. Permutation multivariate analysis of variance (adonis function in R vegan package) based on Bray–Curtis dissimilarities applied on square-root-transformed relative abundances to test the effect of time (fixed, six levels: “T0”, “T1”, “T2”, “T3”, “T4”, “T5”), treatment (fixed, two levels: “ambient”, “heat”) and colony of origin (fixed, three levels: “A”, “B”, “C”) on the coral microbiome. Tank was also fitted in the model. *P*-values were calculated using 10,000 permutations and statistical significance (*p* < 0.05) is shown in bold. Table S16. A linear mixed model (gaussian) testing the effect of treatment (fixed, two levels: “Ambient”, “Heat”) time (fixed, six levels: “T0”, “T1”, “T2”, “T3”, “T4”, “T5”) and colony of origin (fixed, 3 levels) on the Shannon diversity index of the coral microbiome (rarefied data). Tank was included in the model as random effect. Statistical significance (*p* < 0.05) is shown in bold. Table S17 (separate Dataset file). Overview of the 88 highly correlated variables across datasets identified through DIABLO analyses.

## Data Availability

Raw sequence data generated from 16S rRNA gene sequencing and mRNA sequencing is available at the NCBI database under BioProject PRJNA1050834. Coral physiological datasets are available on Research Data Australia [90]. R scripts used for analysing the physiological and omics data are available at https://github.com/emarangon/porites-lutea-responses-to-heat-stress.
