# Supplementary material for: Destabilization of mutualistic interactions shapes the early heat stress response of the coral holobiont
Source: Microbiome. 2025 Jan 31;13:31. doi: 10.1186/s40168-024-02006-5 (PMC11783734; doi:10.1186/s40168-024-02006-5)
Supplement: Supplementary file 2 — Supplementary Material 1. Supplementary figures: Fig S1. Sample clustering for outlier detection in host and Symbiodiniaceae. Heat stress (no; low: < 1 DHW; moderate: 3 DHW) is defined here as ‘stress’. Only samples passing QC are included. Fig S2. Coral respiration rates throughout the heat stress experiment. Mean ± SE is shown over time, and DHW are reported for the heat treatment only (DHW = 0 under ambient conditions). Fig S3. Principal Component Analysis (PCA) of gene expression profiles in host and Symbiodiniaceae on transformed (variance stabilizing transformation) and centered data (left) and multilevel PCA on the same dataset (right) to account for the effect of colony of origin. Fig S4. ASV rarefaction curves of 16S rRNA gene sequences for coral (baseline, T0-T5), seawater and feed (rotifers and microalgae) samples. Fig S5. Non-metric multi-dimensional scaling (NMDS, sqrt-rooted data; stress = 0.17) based on Bray–Curtis dissimilarities calculated on relative abundance of ASVs in coral (Porites lutea), seawater and feed (rotifers and microalgae) samples. Fig S6. Mean relative abundance of dominant microbial families in seawater and feed (rotifers and microalgae) samples. For seawater, the microbial community structure is shown under ambient and heat treatment over time (T0, T3, T5). Fig S7. Changes in relative abundance of the dominant 10 microbial families (> 5% mean relative abundance) across treatments (ambient, heat) and time (baseline, T0—T5) in the coral samples throughout the heat stress experiment. Box = inter-quartile range (IQR), line in box = median, whiskers = minimum and maximum values not outliers (i.e. -/ + 1.5*IQR). Fig S8. Changes in mean relative abundance of differentially abundant ASVs between ambient and heat treatment over time (T0-T5). Differentially abundant ASVs were identified using DESeq (p < 0.01; adjusted post hoc tests), with the restriction that only ASVs present in at least 50% of the samples in one of the compared groups wer [file 40168_2024_2006_MOESM1_ESM.docx]

**SUPPLEMENTARY INFORMATION**

**Destabilization of mutualistic interactions shapes the early heat stress response of the coral holobiont**

Emma Marangon^1,2,3^, Nils Rädecker^4^, Joan Y. Q. Li^2,3^, Marko Terzin^1,2,3^, Patrick Buerger ^5^, Nicole S. Webster^1,6,7^, David G. Bourne^1,2,3^, Patrick W. Laffy^1,3^

^1^ Australian Institute of Marine Science, Townsville, QLD, Australia

^2^ College of Science and Engineering, James Cook University, Townsville, QLD, Australia

^3^ AIMS@JCU, Townsville, QLD, Australia

^4^ Laboratory for Biological Geochemistry, School of Architecture, Civil and Environmental Engineering, École Polytechnique Fédérale de Lausanne, Lausanne, Switzerland

^5^ Applied Biosciences, Macquarie University, North Ryde, NSW, Australia

^6^ Australian Centre for Ecogenomics, University of Queensland, Brisbane, QLD, Australia

^7^ Institute for Marine and Antarctic Studies, University of Tasmania, Hobart, TAS, Australia

**
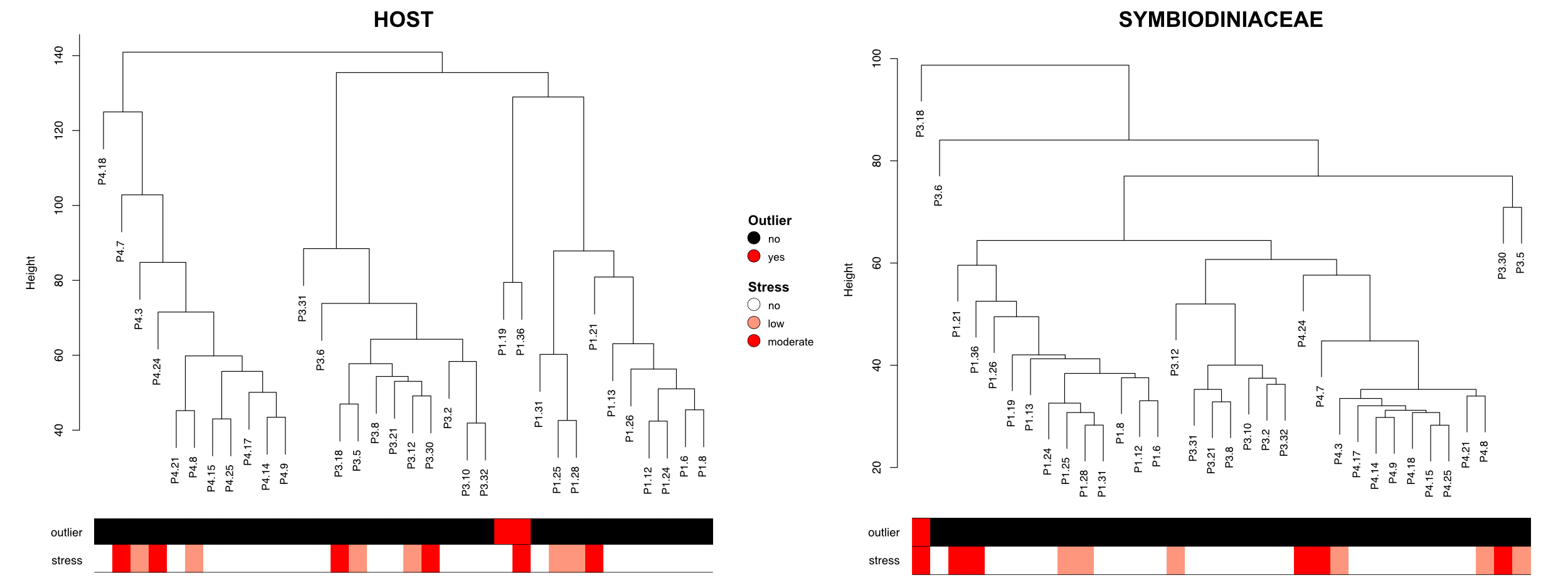
**

**Fig S1**. Sample clustering for outlier detection in host and Symbiodiniaceae. Heat stress (no; low: <1 DHW; moderate: 3 DHW) is defined here as ‘stress’. Only samples passing QC are included.


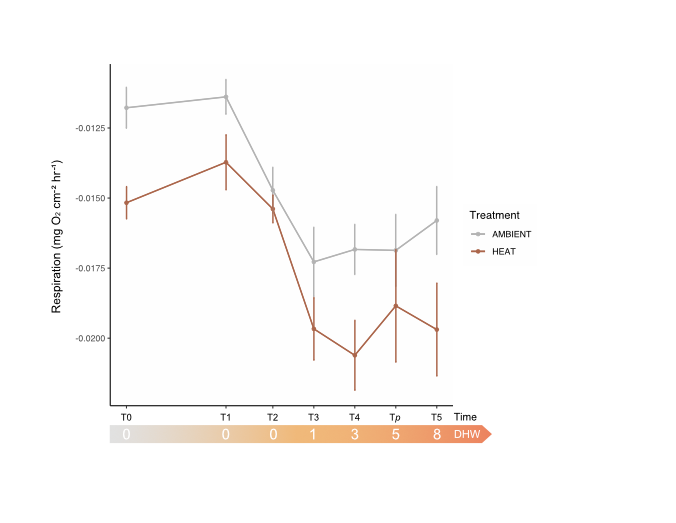


**Fig S2**. Coral respiration rates throughout the heat stress experiment. Mean ± SE is shown over time, and DHW are reported for the heat treatment only (DHW = 0 under ambient conditions).


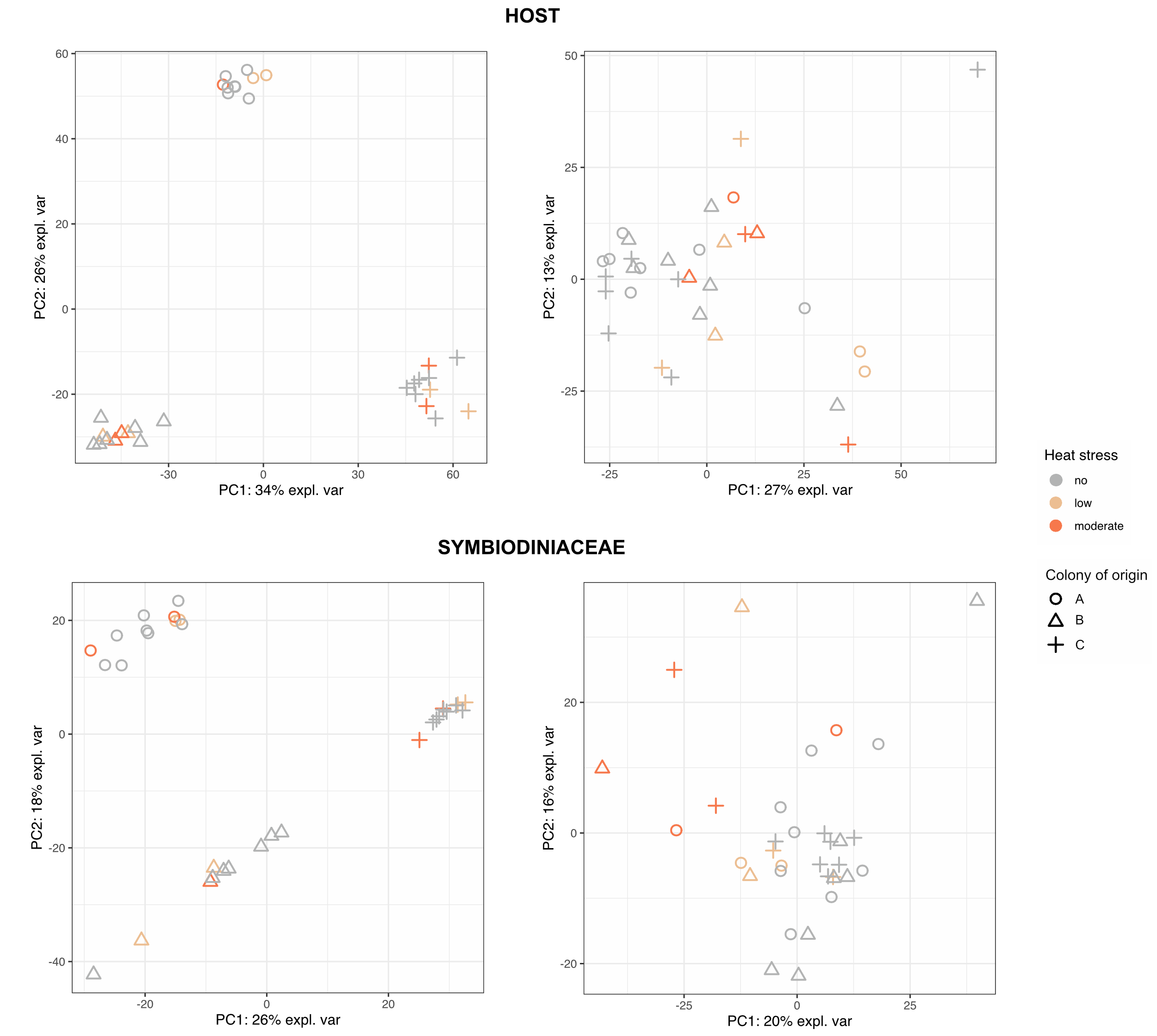


**Fig S3.** Principal Component Analysis (PCA) of gene expression profiles in host and Symbiodiniaceae on transformed (variance stabilizing transformation) and centered data (left) and multilevel PCA on the same dataset (right) to account for the effect of colony of origin.

**
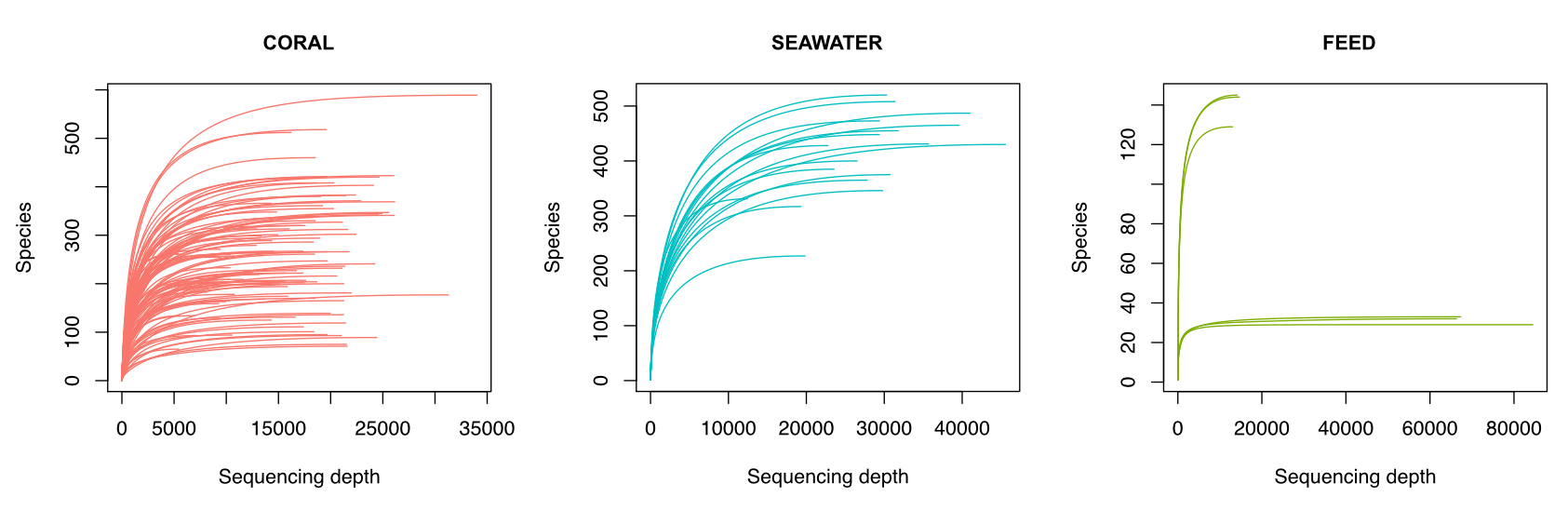
**

**Fig S4.** ASV rarefaction curves of 16S rRNA gene sequences for coral (baseline, T0-T5), seawater and feed (rotifers and microalgae) samples.


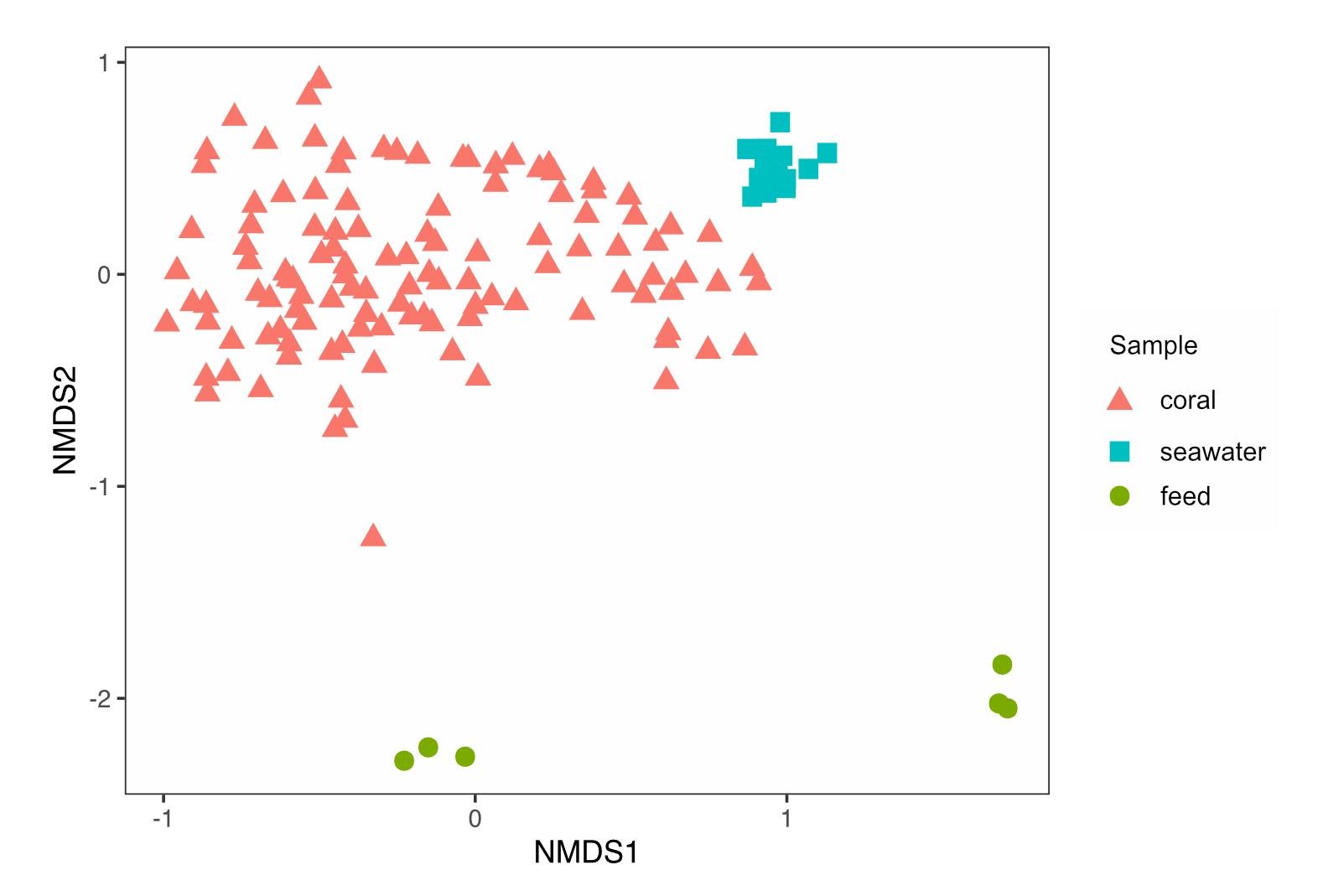


**Fig S5.** Non-metric multi-dimensional scaling (NMDS, sqrt-rooted data; stress = 0.17) based on Bray-Curtis dissimilarities calculated on relative abundance of ASVs in coral (*Porites lutea*), seawater and feed (rotifers and microalgae) samples.


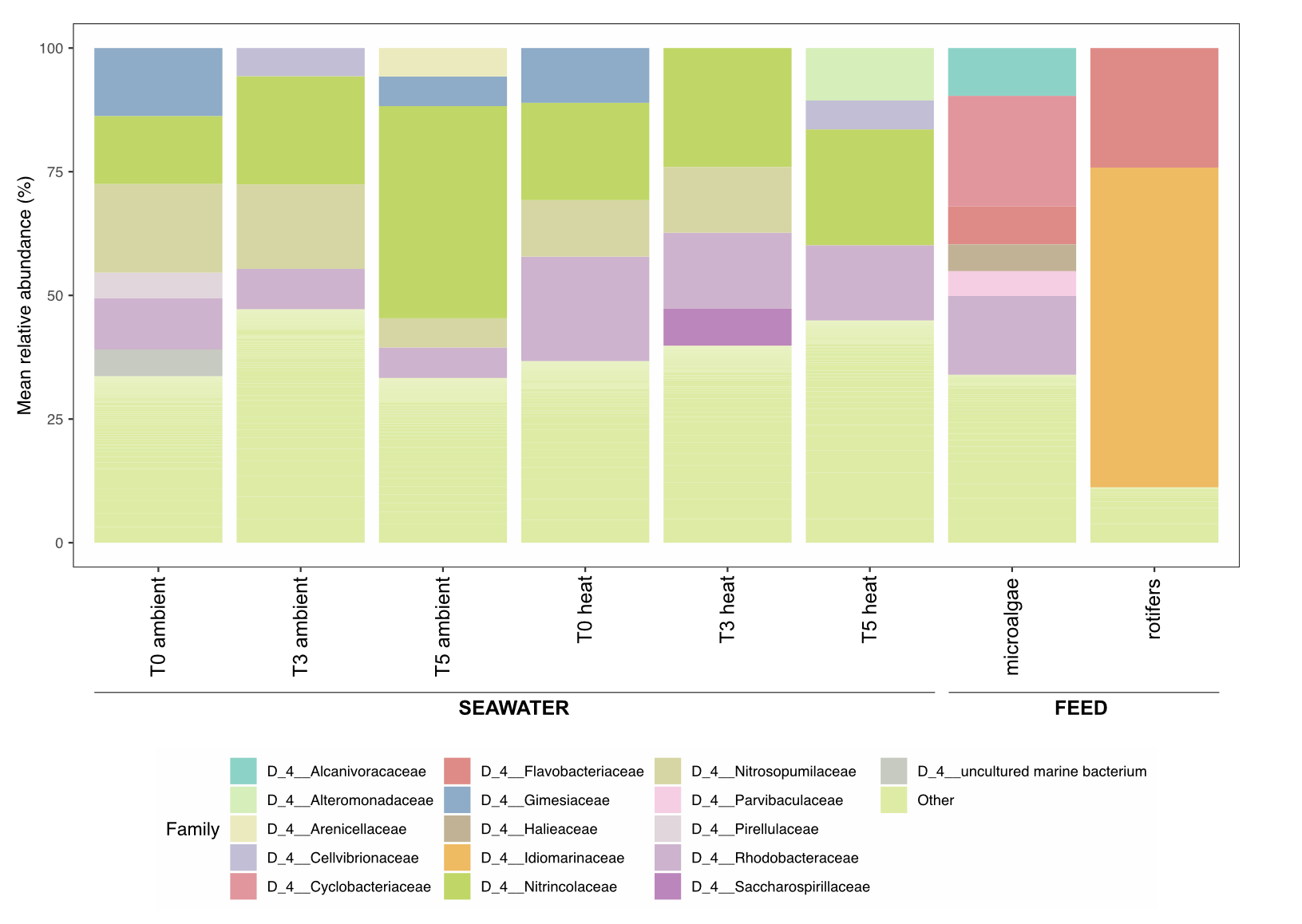


**Fig S6.** Mean relative abundance of dominant microbial families in seawater and feed (rotifers and microalgae) samples. For seawater, the microbial community structure is shown under ambient and heat treatment over time (T0, T3, T5).

**
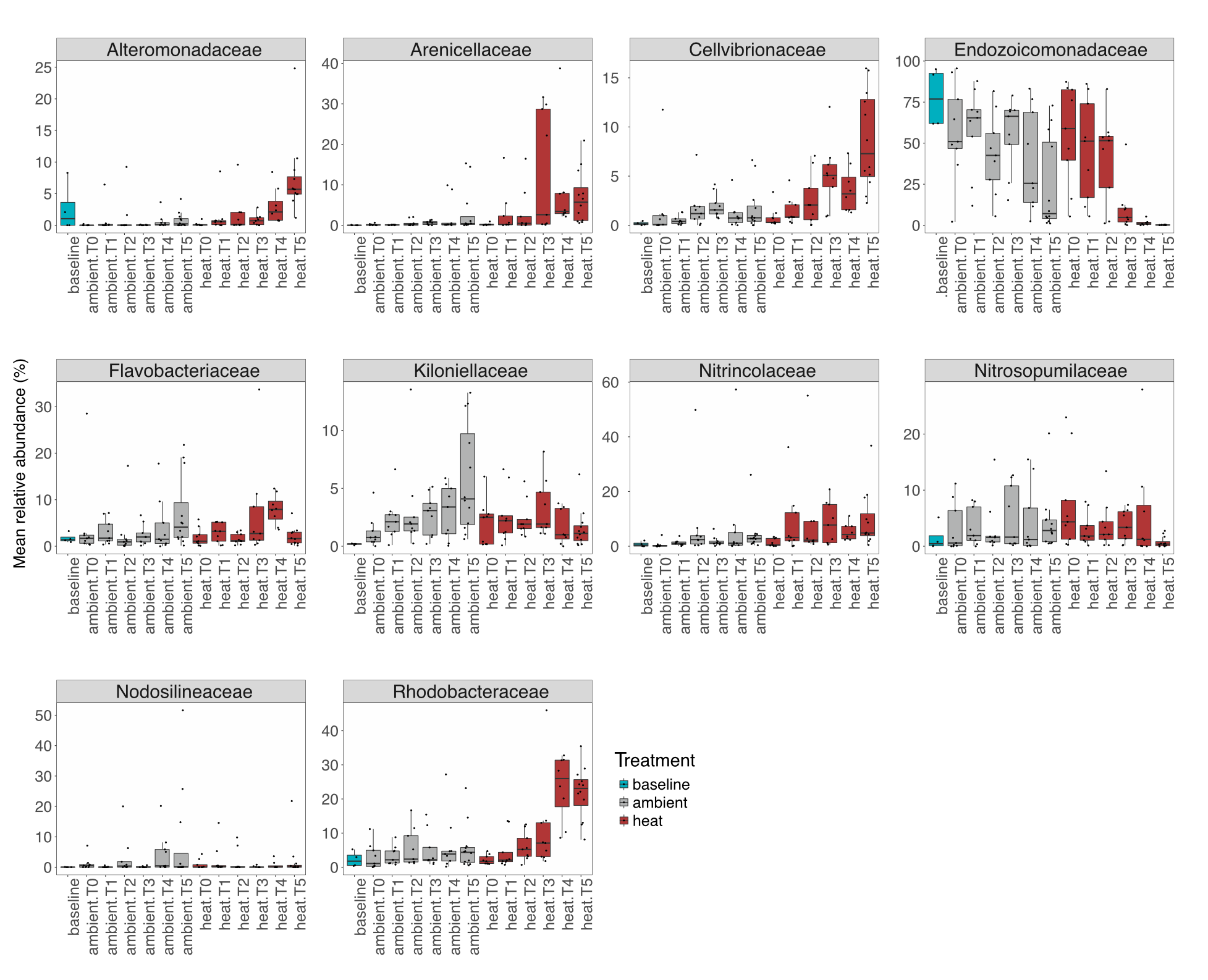
**

**Fig S7.** Changes in relative abundance of the dominant 10 microbial families (> 5 % mean relative abundance) across treatments (ambient, heat) and time (baseline, T0 - T5) in the coral samples throughout the heat stress experiment. Box = inter-quartile range (IQR), line in box = median, whiskers = minimum and maximum values not outliers (i.e. -/+1.5*IQR).


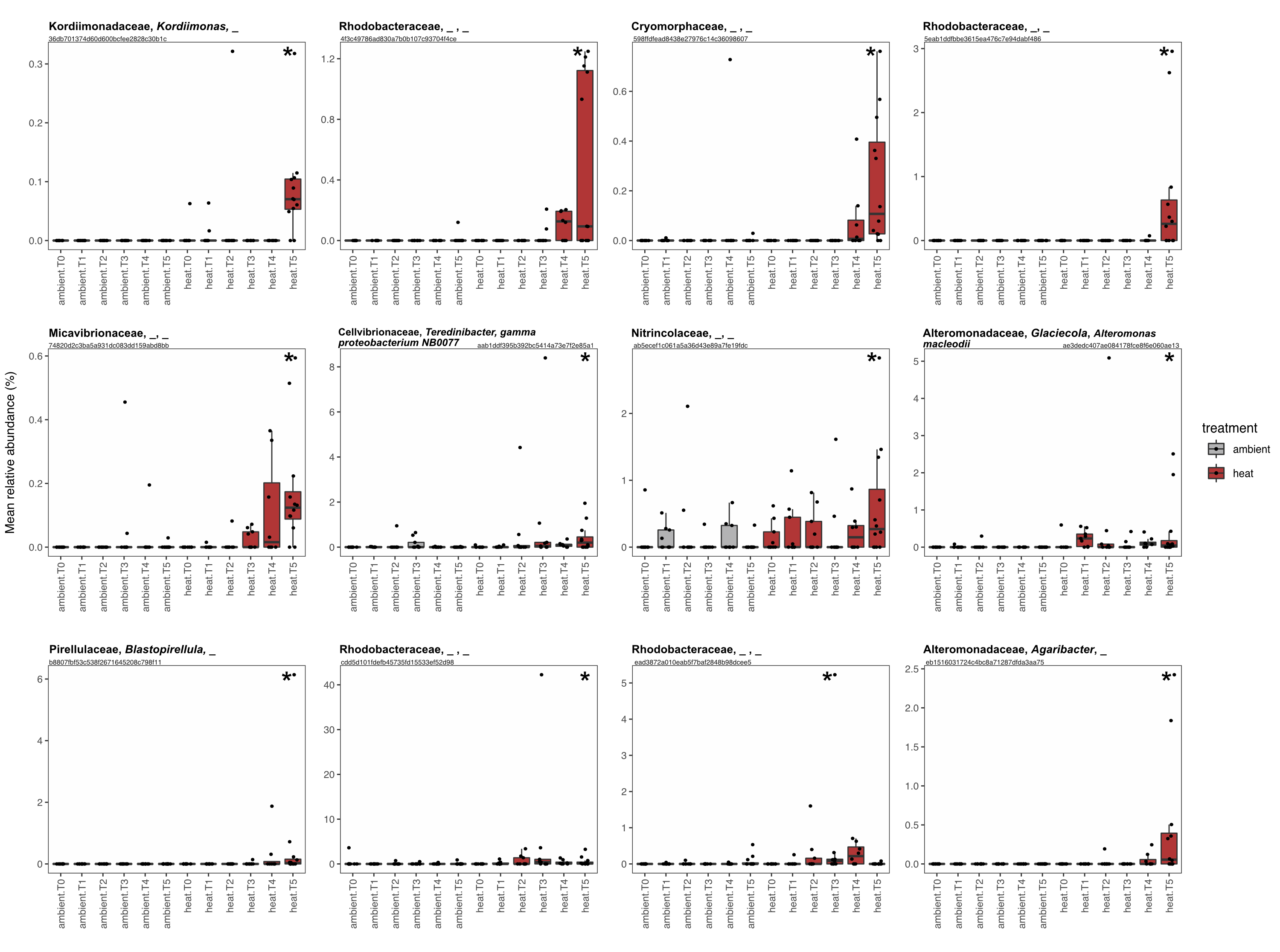


**Fig S8.** Changes in mean relative abundance of differentially abundant ASVs between ambient and heat treatment over time (T0-T5). Differentially abundant ASVs were identified using DESeq (*p* < 0.01; adjusted post hoc tests), with the restriction that only ASVs present in at least 50% of the samples in one of the compared groups were considered. Significant differences are indicated with asterisks (heat vs ambient for the respective time point). Taxonomic assignment for each ASV is shown as Family, Genus, Species. Box = inter-quartile range (IQR), line in box = median, whiskers = minimum and maximum values not outliers (i.e. -/+1.5*IQR).


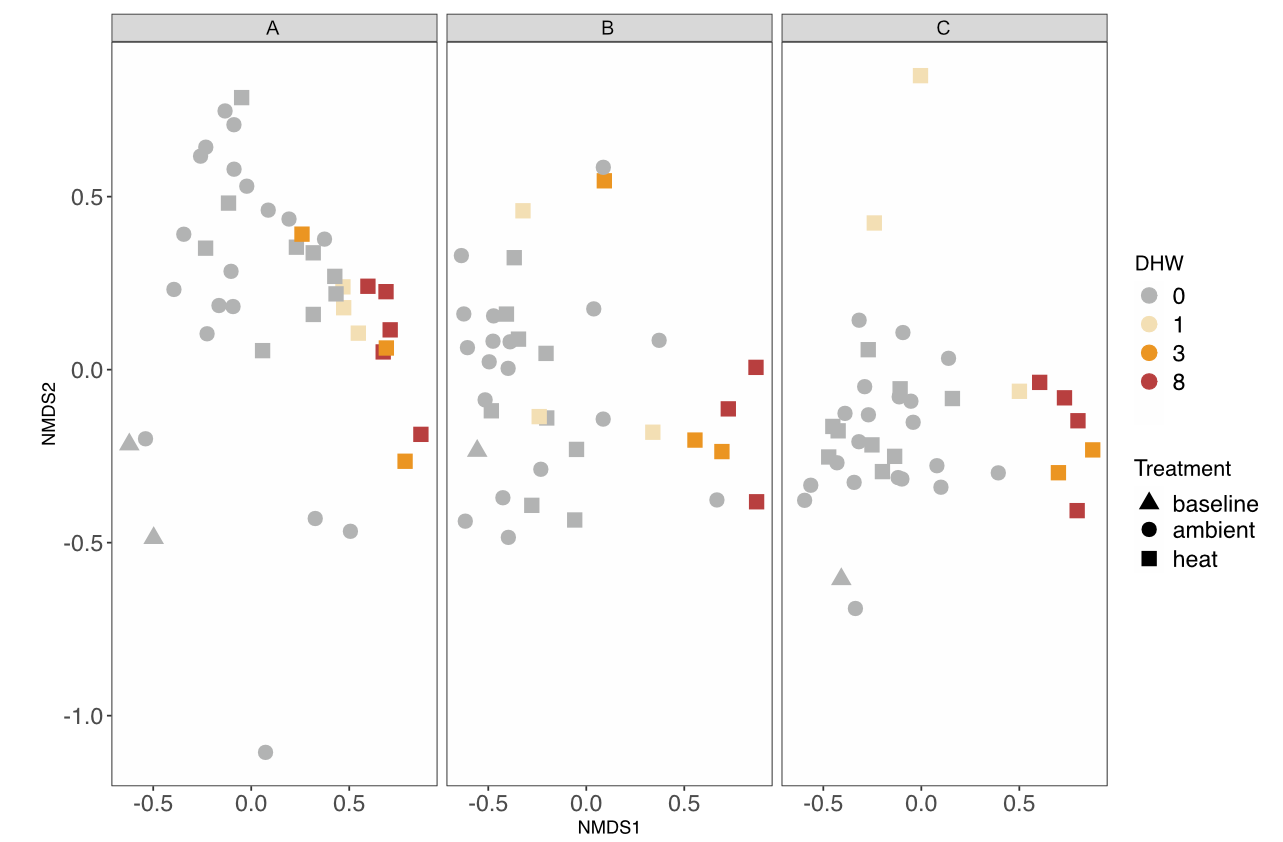


**Fig S9.** NMDS (sqrt-rooted data; stress = 0.21) based on Bray-Curtis dissimilarities calculated on relative abundance of ASVs in coral samples during the heat stress experiment (baseline, ambient, heat) in the three colonies of origin (A, B, C).


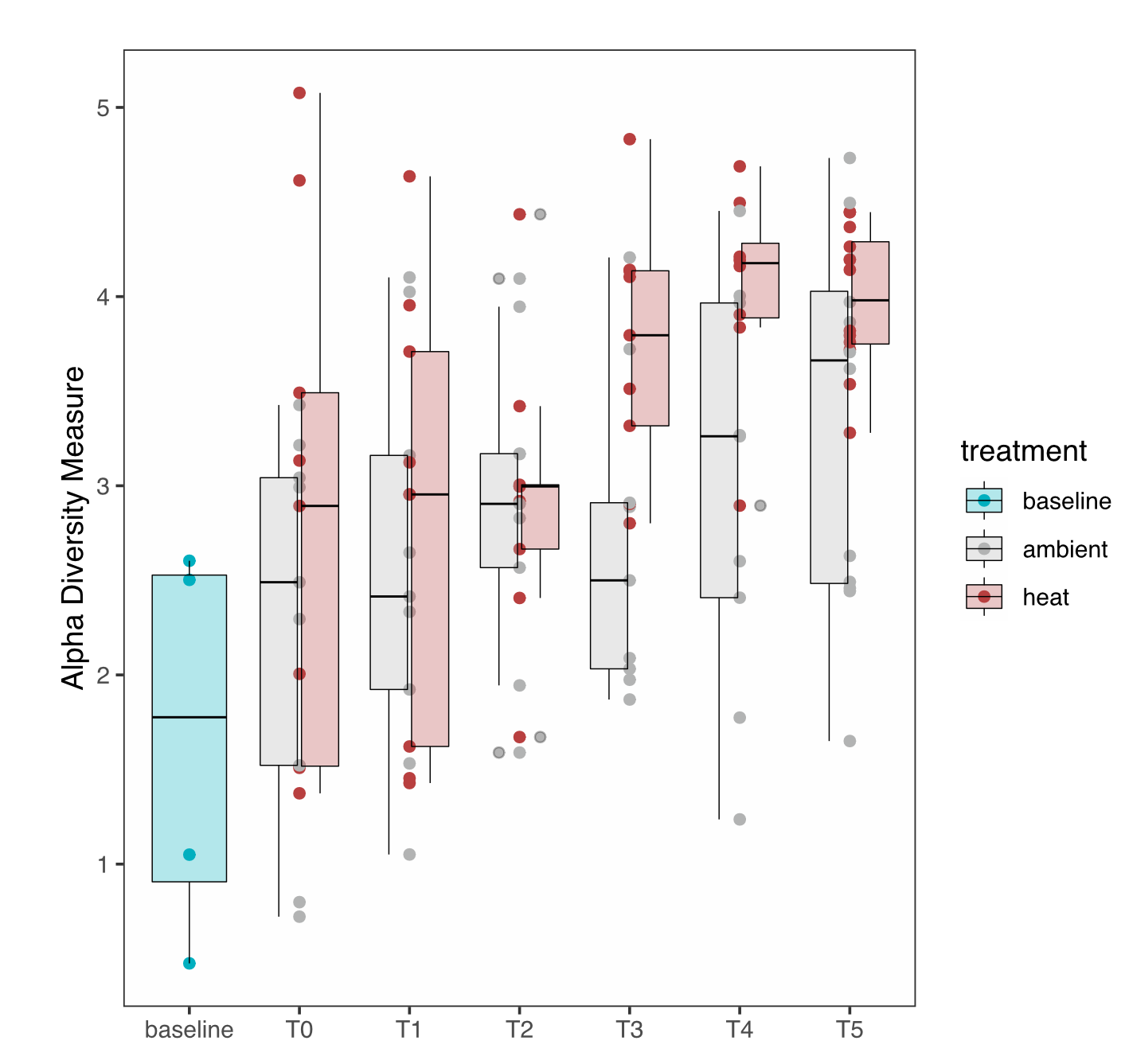


**Fig S10.** Shannon diversity index representing alpha diversity of the microbiome in the coral samples over time (baseline, T0-T5) and across treatments (ambient and heat). Box = inter-quartile range (IQR), line in box = median, whiskers = minimum and maximum values not outliers (i.e. -/+1.5*IQR).


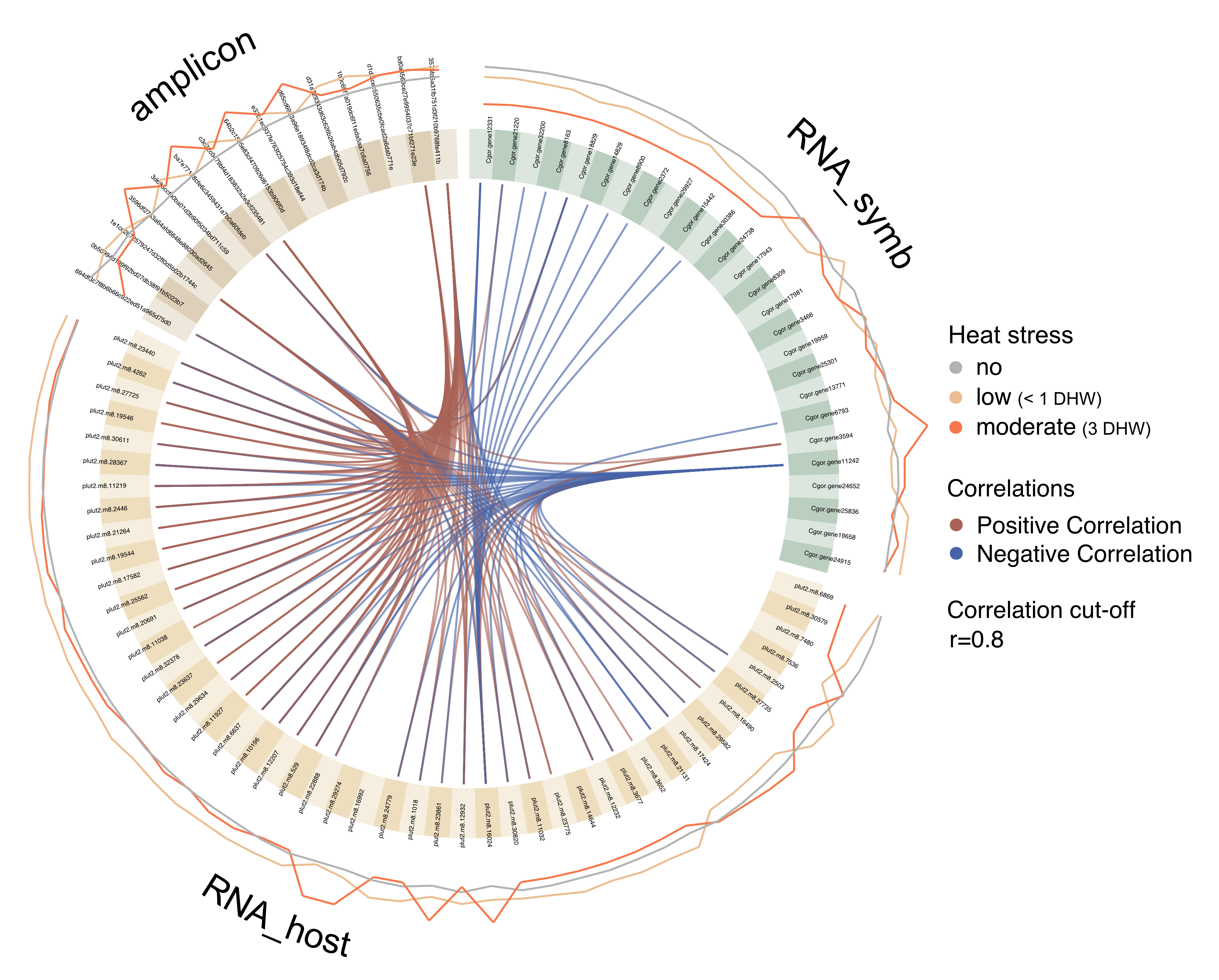


**Fig S11.** The most discriminative features of each omics-dataset along component 1 and 2 (based on DIABLO analyses), and their correlations. The most outer lines represent the abundance level of each variable under ambient, low stress and moderate stress. Features’ taxonomy / functional annotations are outlined in Table S17.

**Table S1.** One ASV was identified as contaminant using the R package decontam (<https://github.com/benjjneb/decontam>).

| **ASV identifier** | **Class** | **Family** | **Genus** |
| --- | --- | --- | --- |
| fd98346394a5c79e554003012cb33826 | Actinobacteria | Micrococcaceae | Renibacterium |

**Table S2.** Generalized linear mixed models tested the fixed effect of treatment (ambient, heat), time (T0, T1, T2, T3, T4, T*p*, T5), colony of origin (A, B, C) and the interaction between treatment and time on: health score, photosynthesis (P; sqrt-transformed), respiration (R; absolute), Gross P:R and photochemical effective efficiency (sqrt-transformed) in the coral *Porites lutea.* Tank and fragment were included in the model to account for repeated measures. The pairwise comparisons (Šidàk correction) for significant interactions are reported here. Estimates are not back-transformed. Significant factors (p<0.05) are indicated in bold.

| *Var.* | *Time Point* | *Contrast* | *Estimate* | *SE* | *df* | *P-value* |
| --- | --- | --- | --- | --- | --- | --- |
| **Health**  **score** | T0 | Ambient-Heat | -1.822 | 0.482 | 442 | **0.001** |
|  | T1 | Ambient-Heat | 0.765 | 0.495 | 442 | 0.544 |
|  | T2 | Ambient-Heat | 0.969 | 0.510 | 442 | 0.303 |
|  | T3 | Ambient-Heat | 0.188 | 0.537 | 442 | 1.000 |
|  | T4 | Ambient-Heat | 2.556 | 0.578 | 442 | **<0.001** |
|  | T5 | Ambient-Heat | 4.142 | 0.670 | 442 | **<0.001** |
| **P** (sqrt) | T0 | Ambient-Heat | -0.002 | 0.006 | 233 | 1.000 |
|  | T1 | Ambient-Heat | -0.006 | 0.006 | 233 | 0.934 |
|  | T2 | Ambient-Heat | 0.011 | 0.006 | 233 | 0.466 |
|  | T3 | Ambient-Heat | 0.017 | 0.006 | 233 | 0.0521 |
|  | T4 | Ambient-Heat | 0.021 | 0.006 | 233 | **0.012** |
|  | T*p* | Ambient-Heat | 0.046 | 0.007 | 233 | **<0.001** |
|  | T5 | Ambient-Heat | 0.090 | 0.007 | 233 | **<0.001** |
| **R** (absolute) | T0 | Ambient-Heat | 18.41 | 5.51 | 233 | **0.007** |
|  | T1 | Ambient-Heat | 14.56 | 5.72 | 233 | 0.079 |
|  | T2 | Ambient-Heat | 3.46 | 5.07 | 233 | 0.992 |
|  | T3 | Ambient-Heat | 6.97 | 4.57 | 233 | 0.618 |
|  | T4 | Ambient-Heat | 9.43 | 4.57 | 233 | 0.251 |
|  | T*p* | Ambient-Heat | 4.19 | 5.05 | 233 | 0.974 |
|  | T5 | Ambient-Heat | 10.19 | 5.10 | 233 | 0.285 |
| **P/R ratio** | T0 | Ambient-Heat | -0.118 | 0.045 | 233 | 0.062 |
|  | T1 | Ambient-Heat | -0.014 | 0.052 | 233 | 1.000 |
|  | T2 | Ambient-Heat | -0.117 | 0.061 | 233 | 0.330 |
|  | T3 | Ambient-Heat | -0.216 | 0.071 | 233 | **0.017** |
|  | T4 | Ambient-Heat | -0.310 | 0.075 | 233 | **<0.001** |
|  | T*p* | Ambient-Heat | -0.500 | 0.097 | 233 | **<0.001** |
|  | T5 | Ambient-Heat | -1.358 | 0.138 | 233 | **<0.001** |
| **Phot.**  **Efficiency** (sqrt) | T0 | Ambient-Heat | 0.039 | 0.038 | 356 | 0.889 |
|  | T1 | Ambient-Heat | 0.055 | 0.040 | 356 | 0.681 |
|  | T2 | Ambient-Heat | 0.090 | 0.040 | 356 | 0.153 |
|  | T3 | Ambient-Heat | 0.130 | 0.045 | 356 | **0.025** |
|  | T4 | Ambient-Heat | 0.202 | 0.049 | 356 | **<0.001** |
|  | T5 | Ambient-Heat | 0.528 | 0.057 | 356 | **<0.001** |

**Table S3. (separate Dataset file).** Summary of sequencing statistics of the transcriptomic dataset, and percentage of reads mapping to the reference genomes.

**Table S4. (separate Dataset file).** Outcome of the Gene Ontology (GO) enrichment analyses (Biological Processes) for the coral host between low stress (< 1 DHW) and ambient conditions; only significant genes (adjusted p < 0.05) are shown.

**Table S5. (separate Dataset file).** Outcome of the Gene Ontology (GO) enrichment analyses (Biological Processes) for the coral host between moderate stress (3 DHW) and ambient conditions; only significant genes (adjusted p < 0.05) are shown.

**Table S6. (separate Dataset file).** Overview of the coral host Differentially Expressed Genes (DEGs) detected between moderate stress (3 DHW) and ambient conditions.

**Table S7. (separate Dataset file).** Overview of the coral host gene expression under moderate stress (3 DHW) compared to ambient conditions, shown as log-fold-changes (logFC). The dataset excludes Differentially Expressed Genes (DEGs), as listed in Table S6.

**Table S8. (separate Dataset file).** Overview of the discriminative coral host genes selected through multilevel sPLS-DA analysis.

**Table S9. (separate Dataset file).** Outcome of the Gene Ontology (GO) enrichment analyses (Biological Processes) for the coral-associated Symbiodiniaceae between low stress (< 1 DHW) and ambient conditions; only significant genes (adjusted p < 0.05) are shown.

**Table S10. (separate Dataset file).** Outcome of the Gene Ontology (GO) enrichment analyses (Biological Processes) for the coral-associated Symbiodiniaceae between moderate stress (3 DHW) and ambient conditions; only significant genes (adjusted p < 0.05) are shown.

**Table S11. (separate Dataset file).** Overview of Symbiodiniaceae Differentially Expressed Genes (DEGs) detected between low stress (< 1 DHW) and ambient conditions.

**Table S12. (separate Dataset file).** Overview of Symbiodiniaceae Differentially Expressed Genes (DEGs) detected between moderate stress (3 DHW) and ambient conditions.

**Table S13. (separate Dataset file).** Overview of the discriminative Symbiodiniaceae genes selected through multilevel sPLS-DA analysis.

**Table S14.** Permutation multivariate analysis of variance (adonis function in R vegan package) based on Bray-Curtis dissimilarities applied on square-root-transformed relative abundances to examine the effects of sample type (fixed, three levels: “coral”, “seawater”, “feed”) on the microbiome. P-values were calculated using 10,000 permutations and statistical significance (*p* < 0.05) is shown in bold.

| *Source* | *df* | *SS* | *F* | *P-value* |
| --- | --- | --- | --- | --- |
| Sample type | 2 | 7.99 | 13.77 | **<0.001** |
| Residual | 138 | 40.04 |  |  |
|  | | | | |
| *Pairwise tests:*  Sample type:  Coral ≠ Seawater  Coral ≠ Feed  Seawater ≠ Feed | | | | |

**Table S15.** Permutation multivariate analysis of variance (adonis function in R vegan package) based on Bray-Curtis dissimilarities applied on square-root-transformed relative abundances to test the effect of time (fixed, six levels: “T0”, “T1”, “T2”, “T3”, “T4”, “T5”), treatment (fixed, two levels: “ambient”, “heat”) and colony of origin (fixed, three levels: “A”, “B”, “C”) on the coral microbiome. Tank was also fitted in the model. P-values were calculated using 10,000 permutations and statistical significance (*p* < 0.05) is shown in bold.

| *Source* | *df* | *SS* | *F* | *P-value* |
| --- | --- | --- | --- | --- |
| Time | 5 | 3.30 | 2.69 | **<0.001** |
| Treatment | 1 | 1.30 | 5.28 | **<0.001** |
| Colony | 2 | 3.87 | 7.87 | **<0.001** |
| Tank | 4 | 1.63 | 1.66 | **<0.001** |
| Time x Treatment | 5 | 1.98 | 1.61 | **<0.001** |
| Residual | 95 | 23.33 |  |  |
|  | | | | |
| *Pairwise tests:*  Colony of origin:  A ≠ B ≠ C  Time x Treatment:  *For time:*  Ambient: T0 ≠ T5  Heat: T0 ≠ T3, T4, T5; T1 ≠ T4, T5; T2 ≠ T4, T5; T3 ≠ T5, T4 ≠ T5  *For treatment:*  T0: ns  T1: ns  T2: ns  T3: Ambient ≠ Heat  T4: Ambient ≠ Heat  T5: Ambient ≠ Heat | | | | |

**Table S16**. A linear mixed model (gaussian) testing the effect of treatment (fixed, two levels: “Ambient”, “Heat”), time (fixed, six levels: “T0”, “T1”, “T2”, “T3”, “T4”, “T5”) and colony of origin (fixed, 3 levels) on the Shannon diversity index of the coral microbiome (rarefied data). Tank was included in the model as random effect. Statistical significance (*p* < 0.05) is shown in bold.

| *Source* | *Estimate* | *SE* | *P-value* |
| --- | --- | --- | --- |
| T1 | 0.30 | 0.38 | 0.433 |
| T2 | 0.63 | 0.38 | 0.095 |
| T3 | 0.41 | 0.38 | 0.281 |
| T4 | 0.72 | 0.38 | 0.059 |
| T5 | 1.12 | 0.36 | **0.002** |
| Heat | 0.57 | 0.40 | 0.155 |
| Colony B | -0.35 | 0.19 | 0.059 |
| Colony C | -0.69 | 0.18 | **<0.001** |
| T1:heat | -0.27 | 0.54 | 0.611 |
| T2:heat | -0.53 | 0.54 | 0.321 |
| T3:heat | 0.47 | 0.54 | 0.381 |
| T4:heat | 0.43 | 0.55 | 0.428 |
| T5:heat | -0.02 | 0.50 | 0.970 |

**Table S17 (separate Dataset file).** Overview of the 88 highly correlated variables across datasets identified through DIABLO analyses.
